# Transplanted human intestinal organoids: a resource for modeling human intestinal development

**DOI:** 10.1242/dev.201416

**Published:** 2023-05-05

**Authors:** Akaljot Singh, Holly M. Poling, Praneet Chaturvedi, Konrad Thorner, Nambirajan Sundaram, Daniel O. Kechele, Charlie J. Childs, Heather A. McCauley, Garrett W. Fisher, Nicole E. Brown, Jason R. Spence, James M. Wells, Michael A. Helmrath

**Affiliations:** ^1^ Division of Pediatric General and Thoracic Surgery, Cincinnati Children's Hospital Medical Center, Cincinnati, OH 45229, USA; ^2^ Division of Developmental Biology, Cincinnati Children's Hospital Medical Center, Cincinnati, OH 45229, USA; ^3^ Center for Stem Cell and Organoid Medicine, Cincinnati Children's Hospital Medical Center, Cincinnati, OH 45229, USA; ^4^ Department of Cell and Developmental Biology, University of Michigan Medical School, Ann Arbor, MI 48109, USA

**Keywords:** Organoid, Intestine, Human, Fetal

## Abstract

The *in vitro* differentiation of pluripotent stem cells into human intestinal organoids (HIOs) has served as a powerful means for creating complex three-dimensional intestinal structures. Owing to their diverse cell populations, transplantation into an animal host is supported with this system and allows the temporal formation of fully laminated structures, including crypt-villus architecture and smooth muscle layers that resemble native human intestine. Although the endpoint of HIO engraftment has been well described, here we aim to elucidate the developmental stages of HIO engraftment and establish whether it parallels fetal human intestinal development. We analyzed a time course of transplanted HIOs histologically at 2, 4, 6 and 8 weeks post-transplantation, and demonstrated that HIO maturation closely resembles key stages of fetal human intestinal development. We also utilized single-nuclear RNA sequencing to determine and track the emergence of distinct cell populations over time, and validated our transcriptomic data through *in situ* protein expression. These observations suggest that transplanted HIOs do indeed recapitulate early intestinal development, solidifying their value as a human intestinal model system.

## Teaser

**Summary:** Histological and transcriptomic comparison of transplanted human intestinal organoids and human fetal intestinal tissue at different developmental stages reveals that these organoids are an effective tool for modeling human intestinal development.

## INTRODUCTION

Insight into intestinal development and disease has traditionally been gleaned from non-human organisms (Zorn and Wells, 2009; Spence et al., 2011a). Unfortunately, some aspects of development and disease vary between organisms. For example, whereas murine crypt development occurs postnatally (Chin et al., 2017), in humans it occurs at the end of the first trimester (Grand et al., 1976). Additionally, the mechanisms underlying villus morphogenesis differ between species (Walton et al., 2012, 2018; Shyer et al., 2013, 2015). The existence of human-specific congenital diseases, such as diarrhea-9 (O'Connell et al., 2018), further highlight the need for understanding human intestinal development.

Until recently, it was challenging to interrogate human intestinal development. Access to fetal human intestine is limited, and its use is subject to legal regulations. Furthermore, human fetal tissue cannot be experimentally manipulated. Thus, we developed human intestinal organoids (HIOs), experimentally tractable models of fetal human intestine, from pluripotent stem cells (PSCs) (Spence et al., 2011b). Unlike crypt-derived enteroids, HIOs contain both epithelium and mesenchyme (Spence et al., 2011b; Watson et al., 2014). After transplantation into immunocompromised mice for 8 weeks, transplanted HIOs (tHIOs) develop a robust crypt/villus axis and enteric smooth muscle (Watson et al., 2014). However, it is unknown whether tHIO development mimics fetal intestinal formation. Teasing this out is integral for determining whether tHIOs model fetal development and disease.

The ability to evaluate epithelial development and mesenchymal differentiation temporally is a major advantage of HIOs that may provide new insight into the formation of the human intestinal niche (Pinchuk et al., 2010; Powell et al., 2011). Recently, interest in a specific fibroblast cell, the telocyte, has arisen because it helps maintain the murine intestine epithelium at homeostasis (Aoki et al., 2016; Shoshkes-Carmel et al., 2018). However, the identities and functions of other human mesenchymal populations are poorly understood. Several recent studies have begun cataloging mesenchymal cells via single-cell RNA sequencing (scRNA-seq) of fetal intestine (Elmentaite et al., 2020; Fawkner-Corbett et al., 2021; Holloway et al., 2021; Yu et al., 2021). Unfortunately, these dissociation strategies do not work well on postnatal human tissue and tHIOs because of the difficulty of extracting mesenchymal cells from the extracellular matrix. To interrogate cellular identity in other complex tissues, researchers have established protocols for single-nucleus RNA sequencing (snRNA-seq), which allows for extraction and transcriptional identification of cell types that are difficult to isolate (Grindberg et al., 2013; Ding et al., 2020). To our knowledge, snRNA-seq protocols have not yet been developed for full-thickness human intestine.

We hypothesize that tHIO maturation post-transplantation is like fetal human intestinal development. To test our hypothesis, we compared a timed developmental study of tHIOs with human intestinal development at morphological, transcriptomic and protein expression levels. We used morphological changes and staining to establish that the tHIOs underwent the same developmental stages as human intestine. Then, we fashioned an snRNA-seq protocol to catalog tHIO development. We compared this dataset with single-cell fetal datasets to determine the cell types in tHIOs. Thus, we highlight the use of tHIOs as a resource for studying fetal human intestinal development.

## RESULTS AND DISCUSSION

**Fig. 1.**
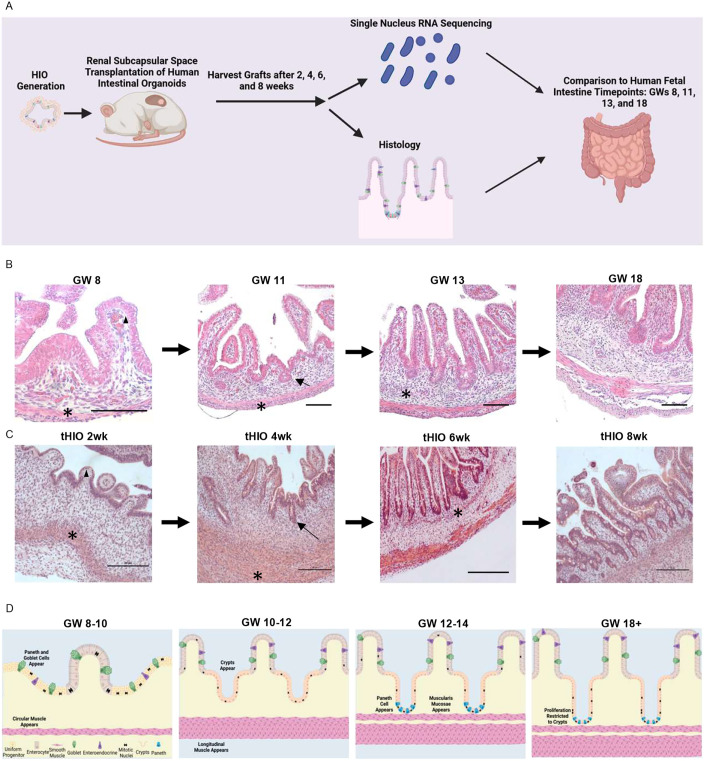
**tHIO development mimics human fetal intestinal development.** (A) Schematic showing the experimental design. (B,C) H&E staining of fetal human intestine over time (*n*=1 per time point) (B) and of tHIOs over time (*n*=5 per time point) (C). Arrowheads indicate villus formation, asterisks indicate formation of a new layer of muscle, and arrows indicate crypt formation. Circular muscle forms at GW 8 in fetal tissue and at 2 weeks in tHIOs, longitudinal muscle forms at GW 11 and tHIO 4 weeks, and muscularis mucosae forms at GW 13 and tHIO 6 weeks. Scale bars: 50 µm in B for GW8; 100 µm in B for GW11, GW13 and GE18; 50 µm in C. (D) Summary schematics of human intestinal development.

**Fig. 2.**
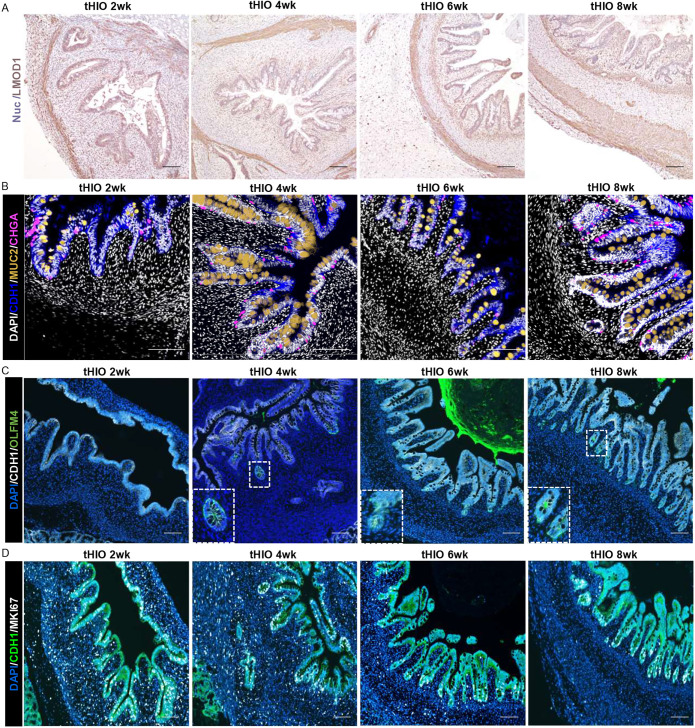
**The tHIO system can be used to study cellular development.** (A) Mayer's Hematoxylin (Lillie's Modification) staining for enteric muscle (LMOD1 immunostaining, brown). Nuc, nuclei. (B) Immunostaining for goblet cells (MUC2, gold), enteroendocrine cells (CHGA, pink) and epithelium (CDH1, blue). (C) Immunostaining for crypts (OLFM4, green) and epithelium (CDH1, white). (D) Immunostaining for proliferation (MKI67, white) and epithelium (CDH1, green), shows restriction of proliferation to the crypts by 8 weeks. *n*=5 per time point. DAPI (white in B, blue in C,D) was used to stain nuclei. Boxed areas in C are shown at higher magnification in insets. Scale bars: 50 µm in A; 100 µm in B-D.

To determine whether tHIO morphological development is comparable to human intestinal development, we evaluated tHIOs histologically every 2 weeks post-transplantation (Fig. 1A). We used the literature to develop staging criteria for the grafts and verified our findings with fetal intestine Hematoxylin & Eosin (H&E) staining. Between gestational week (GW) 8 and 10, the circular muscle layer forms (Fig. 1B,D) (Wallace and Burns, 2005; Liu et al., 2022). Additionally, the pseudostratified epithelium begins to become columnar (Elmentaite et al., 2020), coinciding with goblet and enteroendocrine cell differentiation (Montgomery et al., 1999; Fawkner-Corbett et al., 2021). We observed similar morphology in the 2-week tHIO, including: development of rudimentary villi, loss of pseudostratified cells and formation of circular muscle (Fig. 1C), as assessed through leiomodin 1 (LMOD1) staining (Halim et al., 2017) (Fig. 2A), and emergence of goblet and enteroendocrine cells, as assessed through mucin 2 (MUC2) (Tytgat et al., 1994) and chromogranin A (CHGA) staining, respectively (O'Connor et al., 1983) (Fig. 2B).

Between GW 10 and 12, longitudinal muscle and intestinal crypts form (Fig. 1B,D) (Moxey and Trier, 1978; Montgomery et al., 1999; Wallace and Burns, 2005; Holloway et al., 2021; Liu et al., 2022). These findings were replicated at 4 weeks, when longitudinal muscle (Figs 1C and 2A) and crypt formation were observed, as assessed through expression of the crypt-cell marker olfactomedin 4 (OLFM4) (Fig. 2C) (Burclaff et al., 2022).

Between GW 12 and 14, muscularis mucosae is formed, and Paneth cells appear (Fig. 1B,D) (Moxey and Trier, 1978; Montgomery et al., 1999; Wallace and Burns, 2005; Lueschow and McElroy, 2020; Fawkner-Corbett et al., 2021; Holloway et al., 2021; Liu et al., 2022). Similarly, we observed the formation of muscularis mucosae (Figs 1D and 2A) along with expression of the Paneth cell marker defensin α5 (DEFA5) (Zhao et al., 1996) (Fig. S1A) at 6 weeks.

Finally, at GW 18, proliferation is confined to the crypts (Fig. 1D; Fig. S2) (Montgomery et al., 1999). Staining for marker of proliferation Ki-67 (MKI67) (Basak et al., 2014) revealed heavy proliferation in both epithelium and mesenchyme at 2 weeks and 4 weeks (Fig. 2D). However, by 8 weeks, proliferation was restricted to the crypts. Thus, tHIOs demonstrate similar morphological and cellular maturation patterns to fetal intestinal epithelium.

We further used immunohistochemistry (IHC) and immunofluorescence (IF) to interrogate cellular maturation. The enterocyte brush border marker sucrase isomaltase (SI) (Montgomery et al., 1999), was present at all time points (Fig. S1B). However, staining for alkaline phosphatase (ALPI) (Goldstein et al., 1982) activity revealed faint expression beginning at 4 weeks, with increasing activity at 6 weeks and 8 weeks (Fig. S1C). Likewise, staining for fatty acid binding protein 2 (FABP2) (Storch and Corsico, 2008), which facilitates fatty acid transport, was first identified at 6 weeks in the villus tips (Fig. S1D). Expression spread throughout the villus by 8 weeks. These findings suggest that, although cellular identity is established early, tHIO cells mature at later time points. Thus, tHIOs are excellent for probing cellular maturation.

**Fig. 3.**
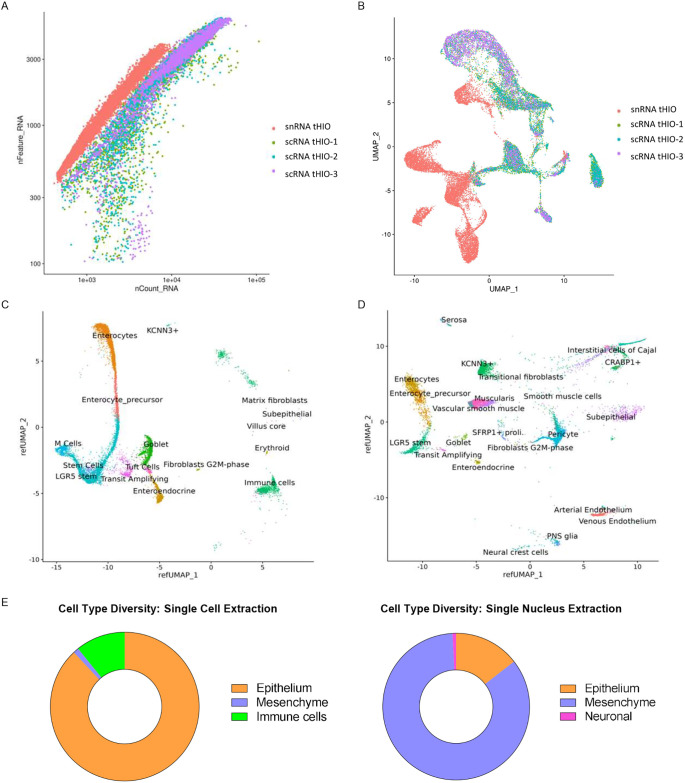
**Comparison of scRNA-seq and snRNA-seq in mature tHIOs.** (A) Comparison of the number of genes and transcripts identified in each cell/nucleus. (B) Merged UMAP projection of cells isolated from each technique. (C) Cell types identified by scRNA-seq. (D) Cell types identified by snRNA-seq. (E) Frequency of each umbrella cell type obtained from the respective extraction protocols. *n*=3 (scRNA sequencing); *n*=1 (snRNA sequencing).

To determine whether scRNA-seq or snRNA-seq was more suitable for capturing the most diverse set of cells, we compared data generated from the two methods on tHIOs harvested at a minimum of 8 weeks post-transplantation (Fig. 3). The single-nucleus dataset contained 12,463 nuclei, and the single-cell dataset contained 16,743 cells. The cells expressed twice as many genes (∼3086 genes/cell), on average, as did the nuclei (∼1562 genes/nucleus). However, more genes were found for a given number of reads in the nuclei than in the cells (Fig. 3A), suggesting that snRNA-seq was more efficient at mapping reads. The datasets did not integrate well (Fig. 3B), likely because of both differences in read depth as well as the presence of cell populations not detected by scRNA-seq. An alternative strategy was required to determine cell identity.

Because many of the classical adult intestinal cell markers were not found in our snRNA-seq dataset, we used machine learning to identify cell populations. We constructed a reference atlas using three publicly available human fetal intestinal scRNA-seq datasets (Fig. S3A) (Elmentaite et al., 2020; Fawkner-Corbett et al., 2021; Thorner et al., 2021 preprint; Yu et al., 2021). Next, we used Seurat's ‘Map Query Projection’ function to project our cells onto the reference atlas (Fig. 3C,D). The scRNA-seq tHIO dataset consisted of 90.81% epithelial cells, 8.24% immune cells and 0.86% mesenchymal cells, heavily favoring epithelial populations. Only five mesenchymal lineages were detected. No smooth muscle, enteric nerves or endothelial cells were identified (Fig. 3E). In contrast, the snRNA-seq tHIO dataset comprised a more diverse set of cells, including 14.37% epithelial lineages, 84.82% mesenchymal lineages and 0.81% neuronal lineages (Fig. 3F). Fourteen distinct mesenchymal lineages were identified, including smooth muscle. However, ‘M Cells’ and ‘Immune cells’ were not found (Fig. 3C,D). This rich cellular diversity was also found in a full-thickness adult duodenum (Fig. S3B). Taken together, these findings suggest that, whereas scRNA-seq is excellent for isolating epithelium, snRNA-seq is better for extracting non-epithelial populations. We thus used snRNA-seq for the study.

**Fig. 4.**
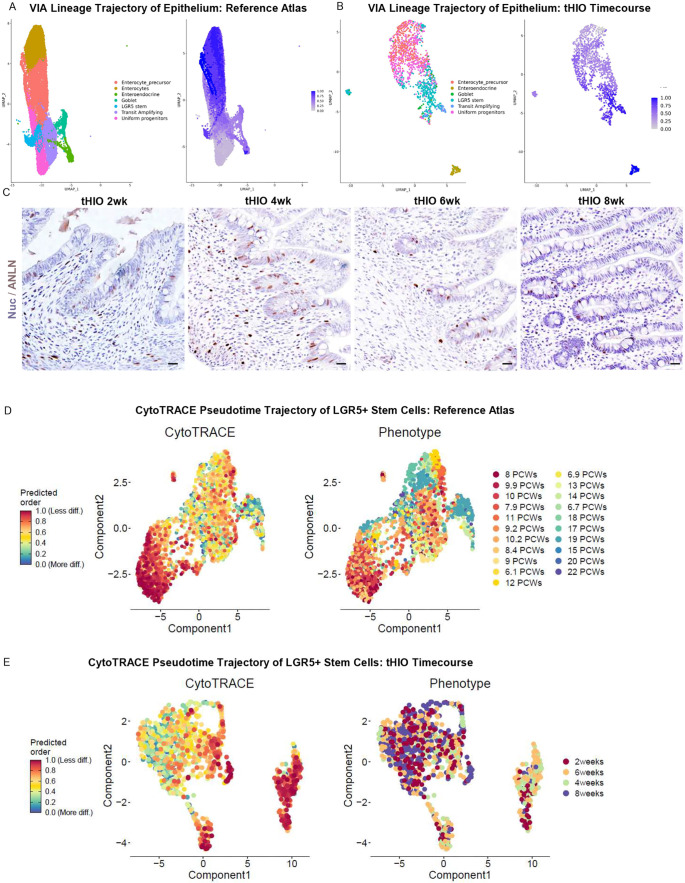
**Epithelial development in tHIOs mimics fetal human intestinal epithelial development.** (A) UMAP and VIA pseudotime trajectory of epithelial cell development in the reference atlas. (B) UMAP and VIA pseudotime trajectory of epithelial cell development in the tHIO time course. (C) Staining for transit-amplifying cells (ANLN immunostaining, brown) (*n*=5 per time point). Nuc, nuclei. (D) CytoTRACE pseudotime trajectory of human fetal LGR5^+^ stem cells. (E) CytoTRACE pseudotime trajectory of tHIO LGR5^+^ stem cells. PCW, post-conception week. Scale bars: 10 μm.

To determine informatically whether tHIOs could be used to investigate epithelial development, we performed a lineage trajectory analysis on both datasets (Fig. 4A,B). The ‘Uniform Progenitor’, an early intestinal stem cell population (Elmentaite et al., 2020), was the least differentiated subtype in both datasets. The trajectory analysis of the reference atlas indicated that this pool generated transit-amplifying cells, which in turn became more mature cell types, including LGR5^+^ stem cells, enterocytes and secretory lineages. Similar results were found in the tHIO time course. The reference atlas suggested anillin (ANLN) as a novel marker of transit-amplifying cells. ANLN staining revealed diffuse epithelial expression at early time points, in line with our trajectory results (Fig. 4C). By 6 weeks, ANLN expression was restricted to the crypts, reminiscent of more mature tissue.

To interrogate whether individual epithelial cell population development could be studied using tHIOs, we performed a head-to-head comparison of the transcriptional trajectory of LGR5^+^ stem cells from both datasets (Gulati et al., 2020). In both datasets, LGR5^+^ stem cell gene expression remained stable over time (Fig. 4D,E). Although there was clear separation of the cells at the first and last time points (Fig. 4E,F), the cells in the middle time points did not progressively change, indicating that the cells were transcriptionally similar over time. Moreover, the upregulated biological processes in the ‘less differentiated’ and ‘more differentiated’ LGR5^+^ stem cells in both datasets were similar (Fig. S4A,B). These findings suggest that the tHIOs are a robust tool for transcriptionally evaluating intestinal epithelial development.

To determine informatically whether tHIOs could be used to investigate mesenchymal development, we performed a lineage trajectory analysis on both datasets (Fig. S5A,B). The ‘Transitional Fibroblast’ population was the least differentiated in the reference atlas (Fawkner-Corbett et al., 2021). This population evolved into the CD81^high^, the KCNN3^+^ and the CCL19^+^ fibroblast lineages, which in turn developed into the serosa, the enteric smooth muscle and the vasculature. The ‘Transitional Fibroblast’ and CCL19^+^ subtypes were not detected in the tHIOs, likely owing to issues with single-nucleus read depth. However, the KCCN3^+^ and CD81^high^ fibroblasts were detected and found to be less differentiated than the enteric smooth muscle, myofibroblasts and serosa, suggesting that the tHIO findings were similar to the reference atlas findings.

To interrogate whether the development of an individual mesenchymal cell population could be studied using tHIOs, we performed a head-to-head comparison of telocyte transcriptional trajectory over time (Fig. S5C,D). In both datasets, telocyte gene expression remained stable over time, as the cells did not progressively delineate over time. Moreover, the upregulated biological processes in the ‘less differentiated’ and ‘more differentiated’ telocytes in both datasets were similar (Fig. S4C,D). These findings suggest that the tHIOs are an excellent tool for transcriptionally evaluating intestinal mesenchyme development.

Because limited data about the mesenchyme exists, few IHC markers have been identified. We thus used our reference atlas to identify putative markers of noteworthy fibroblast populations and traced these lineages over time. In the human intestine, coagulation factor III (F3) specifically marks subepithelial ‘telocytes’ (Kinchen et al., 2018), which support murine intestinal stem cells at homeostasis (Aoki et al., 2016; Shoshkes-Carmel et al., 2018). Weak F3 expression was identified at 2 weeks (Fig. S5E). Stain intensity increased over time, especially along new villi, suggesting that F3^+^ cells could play a role in villification. This hypothesis is further supported by analysis of upregulated telocyte biological processes, which include regulation of cell differentiation and tube development (Fig. S7A).

Next, our reference atlas identified CD81 (also known as tetraspanin-28) as a marker of the CD81^high^ fibroblast population. Interestingly, at the 2 week timepoint, many of the fibroblasts appeared to express CD81. Over time, however, fewer fibroblasts expressed the protein (Fig. S6A). The upregulated biological processes in this lineage included ‘extracellular matrix production’ and ‘collagen fibril organization’ (Fig. S7C), suggesting that this population is involved in matrix production.

A third population of interest was the KCNN3^+^ fibroblast, which was found to express potassium calcium-activated channel subfamily N member 3 (KCNN3) at high levels. This population was present diffusely in the mesenchyme at every time point, but was especially concentrated outside the muscle (Fig. S6B). Whereas the muscularis expressed cytoplasmic KCNN3, KCNN3^+^ fibroblasts expressed nuclear KCNN3. The upregulated biological processes in this lineage included ‘extracellular matrix organization’ and ‘muscle contraction’, and downregulated biological processes included ‘cell division’, ‘cell migration’ and ‘mesenchyme development’ (Fig. S7D). These findings suggest that this lineage may regulate matrix production by other fibroblasts, such as the CD81^high^ lineages. Indeed, another putative KCNN3^+^ fibroblast marker gene, asporin (*ASPN*), has been implicated in the control of matrix production by non-intestinal fibroblasts (Xie et al., 2018; Liu et al., 2021). Additionally, a subpopulation of these cells is believed to help mediate enteric muscle relaxation (Vanderwinden et al., 2002; Kim, 2011).

A fourth population of interest was the myofibroblast, which highly expressed tissue plasminogen activator (PLAT). Staining for this population revealed emergence at 4 weeks (Fig. S6C). Although it is located near the epithelium, similarly to the telocyte, the myofibroblast is a distinct mesenchymal lineage (Duckworth, 2021). The upregulated myofibroblast biological processes included ‘epithelial cell proliferation’ and ‘negative regulation of differentiation’, suggesting that this population may contribute to the stem cell niche (Fig. S7B).

Additionally, we stained for endothelial cell marker cluster of differentiation 31 (CD31; PECAM1), to study vasculature development (Pusztaszeri et al., 2006). Although the vasculature had invaded the mesenchyme by 2 weeks, organization appeared to progress over time, reminiscent of a more mature structure by 6 weeks (Fig. S6D).

To gain further insight into mesenchymal subpopulation function, we performed a receptor-ligand analysis on some of the key epithelial niche factors. In both GW 18 intestine and 8 week tHIO, *WNT2B* was expressed by several mesenchymal populations, including KCNN3^+^ fibroblasts and smooth muscle, but not telocytes (Figs S8, S9). In contrast, *WNT5A* was expressed throughout the mesenchyme, including in telocytes. Surprisingly, the classical WNT2B receptor gene *FZD4* was not expressed by epithelial cells; rather, only *FZD5* was expressed in GW18 epithelium, and *FZD9* was expressed by 8 week tHIO epithelium. Additionally, *ROR2* expression was lacking in GW18 epithelium, but robust in LGR5^+^ stem cells and transit-amplifying cells in 8 week tHIOs, suggesting some differences in signaling between the two systems.

The EGF-family niche factor NRG1 promotes epithelial differentiation (Holloway et al., 2021). In both GW18 human intestine and 8 week tHIO, *NRG1* was expressed by telocytes and CD81^high^ fibroblasts (Fig. S10), with more diffuse expression in the tHIO. In both datasets, expression of the NRG1 receptor gene *ERBB2* was found throughout both compartments, while *ERBB3* expression was restricted to the epithelium.

The murine literature suggests that the HIPPO/YAP pathway helps regulate formation of enteric muscle (Cotton et al., 2017). To gain insight into its role in human intestinal development, we analyzed the expression pattern of HIPPO/YAP pathway components in both datasets. We found robust expression of *YAP1* and *WWTR1* in both fetal intestinal and tHIO mesenchyme at GW18 and 8 weeks (Fig. S11A,B). Additionally, expression of the YAP target genes *CYR61* (*CCN1*) and *CTGF* (*CCN2*) were found throughout the mesenchyme. To verify these findings, we stained for members of the YAP-TAZ complex in both tissue types (Fig. S11C,D). Nuclear localization of the YAP-TAZ complex was especially robust in the enteric muscle, suggesting that the HIPPO/YAP pathway may regulate human enteric muscle differentiation.

One limitation of our work is the heavy reliance on supervised machine learning for cell-type annotation. The reference atlas was constructed using ELeFHAnt, which assigns the best-matching cell type to a query if the true cell type is not found. This can potentially lead to cell types being overlooked and inaccuracies in the dataset, potentially impacting downstream analysis. We attempted to mitigate this by manually checking the labels, although this was challenging for mesenchymal subpopulations, as few known markers exist. Another limitation is that we used a trypsin- and Accutase-based approach to dissociate tHIOs to single cells. It is possible that a collagenase-based approach would be more suited for extracting mesenchyme. Further work would focus on extracting mesenchymal cells using various collagenases and other enzymes that dissociate the extracellular matrix.

Our findings suggest that tHIOs are a proxy for studying the development of the human fetal intestine. They mimic human fetal intestinal development on morphological, transcriptional and proteomic levels. Future efforts will involve gene-editing methods to validate individual mesenchymal populations functionally.

## MATERIALS AND METHODS

### Generation of HIOs

*In vitro* HIOs were derived from the H1 embryonic stem cell (ESC) line (WiCell Research Institute) (NIHhESC-10-0043) as previously described (Spence et al., 2011b; Poling et al., 2018). Cells were tested monthly to ensure freedom from *Mycoplasma* contamination, and cultures that were contaminated by any microbe were never used for differentiation. Briefly, ESCs were cultured in Matrigel (BD Biosciences)-coated plates and supplied with mTESR1 media (STEMCELL Technologies). To generate definitive endoderm (DE), cells were split into single cells with Accutase (STEMCELL Technologies). Cell quantity was assessed using a TC20 Automated Cell Counter (Bio-Rad) and plated at a density of approximately 100,000 cells per well in Matrigel-coated, 24-well plates. ESCs were allowed to proliferate for 2 days prior to treatment with 100 ng/ml of activin A (Cell Guidance Systems) in DE induction media [RPM1 1640, 100× NEAA and 0.2-2% dialyzed fetal calf serum (FCS)]. To generate hindgut spheroids, DE was supplied with hindgut induction media (RPMI 1640, 100× NEAA, 2% dFCS) for 4 days, supplemented with 100 ng/ml FGF4 (R&D Systems) and 3 µM CHIR 99021 (Tocris). After a total of 9 days, spheroids were harvested and replated in 24-well plates in bubbles of Growth Factor Reduced Matrigel at a density of approximately 40 spheroids per well. HIO formation was induced by supplying spheroids with intestinal growth medium (Advanced DMEM/F-12, N2 supplement, B27 supplement, 15 mM HEPES, 2 mM L-glutamine, penicillin-streptomycin) supplemented with 100 ng/ml EGF (R&D Systems), as previously described. Media was changed twice per week. HIOs were replated in fresh Growth Factor Reduced Matrigel after 14 days at a density of one HIO per well and were transplanted on day 28.

### Animal handling

Male and female non-obese diabetic, severe combined immunodeficiency, interleukin-2Rγnull (NSG) *Mus muscularis* between the ages of 8 and 12 weeks were used as a host for the transplantation of the HIOs, as previously described (Watson et al., 2014; Singh et al., 2020). Mouse handling was performed in accordance with the NIH Guide for the Care and Use of Laboratory Animals. Mice were maintained in the pathogen-free vivarium at Cincinnati Children's Hospital Medical Center on an antibiotic chow (275 ppm sulfamethoxazole and 1365 ppm trimethoprim; ‘Test Diet’). Both food and water were provided *ad libitum* throughout the entire course of the study. All animal experiments were performed with the approval of the Institutional Animal Care and Use Committee at the Cincinnati Children's Hospital Medical Center (Building and Rebuilding the Human Gut, Protocol No. 2021-0060).

### Transplantation of human intestinal organoids

After 28 days of *in vitro* maturation, HIOs were transplanted into the renal subcapsular space of NSG mice, as previously described (Watson et al., 2014; Singh et al., 2020). Briefly, each mouse was anesthetized with 2.5% inhaled isoflurane (Butler Schein) and the left flank was shaved and sterilized with isopropyl alcohol and povidine-iodine. Bupivacaine (1 mg/kg) was injected near the incision site as a topical anesthetic. A 1-cm incision was made in the posterior subcostal skin, followed by a 1-cm incision in the retroperitoneal muscle. The kidney was removed from the peritoneal cavity and a pocket was created in the kidney capsule. A single HIO was inserted through the pocket and into the subcapsular space. Next, the kidney was returned to the peritoneal cavity. The peritoneal cavity was flushed with a 2.38 mM solution of piperacillin-tazobactam (AuroMedics). Incisions were closed using a double layer closure technique. After closing the incisions, mice were given a single injection of carprofen (1 mg/ml) for pain management.

### Tissue harvest

Beginning at 2 weeks post-transplantation, mice were humanely euthanized and tHIOs were harvested. Mice were euthanized at 2, 4, 6 and 8 weeks for tissue collection; 8 weeks was selected as the endpoint for tissue collection based on several observations since developing the model (Watson et al., 2014). We have evaluated longer time points, but when the transplants are done in the kidney capsule we have not observed additional maturation of epithelial cell types or additional morphological changes (Bouffi et al., 2023). Important for kidney capsule transplants, the tHIO accumulation of mucus and exfoliated epithelium occurs as they are in a closed system, which then leads to attenuation of the graft beyond this time point. Harvested grafts were photographed alongside a metric ruler, and graft width was analyzed using ImageJ. For each image, graft width was measured in pixels, and a 1-mm measurement on the ruler was used to convert pixels to cm. A portion of each graft was flash-frozen for sequencing and a portion was used for IHC. Five grafts were harvested per time point, across three distinct differentiations, based on historical data regarding the number of grafts needed to detect differences between tHIO groups. No grafts were excluded from downstream analysis. Assignment of grafts to be harvested at specific time points was random. Grafts were numbered according to the mouse log number, such that investigators were unaware of groupings at all points of downstream analysis except analysis of sequencing data. Statistical information is laid out for each individual experiment in this section.

### Antibody information

All primary and secondary antibodies were chosen based on inclusion in previous publications, followed by in-house validation on human intestinal tissue prior to use on experimental samples. Primary and secondary antibodies, their dilutions, host species and catalog numbers are listed in Table S1 for convenience.

### Human subjects

#### Adult duodenum

A surgical sample from a single human subject was used for this study. A portion of the duodenum was removed as part of a procedure for chronic pancreatitis, but otherwise the intestine was healthy. Informed consent was obtained from the patient prior to use of the tissue in our study. Human tissue was de-identified prior to use. Cincinnati Children's Hospital Medical Center's Internal Review Board approved our human subject research.

#### Fetal intestinal samples

As noted by Holloway et al. (2021), ‘normal, de-identified human fetal intestinal tissue was obtained from the University of Washington Laboratory of Developmental Biology. All human tissue used in this work was de-identified and was conducted with approval from the University of Michigan IRB.’ All experiments on fetal intestine were conducted at the University of Michigan.

### Tissue processing, IHC, IF and microscopy

A portion of each tHIO was fixed in 4% paraformaldehyde (PFA) at 4°C overnight, processed, and embedded in paraffin. Tissue blocks were cut into either 5-μm- or 10-μm-thick sections and placed on slides for staining, as previously described (Poling et al., 2018; Singh et al., 2020). Briefly, slides were deparaffinized in xylene, rehydrated, and antigen retrieval was performed. Both antibody incubation steps were performed overnight at 4°C. Dilutions and references for all antibodies used are listed in Table S1. Staining images were captured on a Nikon Eclipse Ti and analyzed using Nikon Elements Imaging Software (Nikon). Functional alkaline phosphatase staining was performed using the Vector Red Substrate Kit, Alkaline Phosphatase (Vector Laboratories), which directly stains for ALPI activity using a chromogenic substrate. During the staining and microscopy processes, the investigator was unaware of which group each graft belonged to.

### Epithelial compartment proliferation quantification

To quantify the amount of proliferation in each compartment, grafts (*n*=5 per time point) at the 4 week, 6 week and 8 week time points were co-stained for CDH1 and MKI67. The 2 week time point was excluded because there are no crypts at this time point. Each cell on the slide that was co-positive for MKI67 and CDH1 was categorized as ‘crypt’ or ‘villus’ based on morphology. The percentage of MKI67^+^/CDH1^+^ cells that were present in the crypt was calculated for each graft. A Brown–Forsythe and Welch ANOVA one-tailed test with a Dunnett T3 test for multiple comparisons was used to assess for differences between groups. The investigator was unaware of group identity during the staining process as well as the quantification process.

### Single nucleus extractions

A portion of each tHIO was flash-frozen in liquid nitrogen and stored at −80°C. Single nucleus extractions were performed on flash-frozen tHIOs using the Minute Detergent Free Single Nucleus Isolation Kit (Invent Biotechnologies). The manufacturer's instructions were followed, with a few modifications. Buffer A was supplemented with 100 U of Protector RNAse Inhibitor (Roche), 1 μM DTT (Invitrogen) and 8×10^−5^ M leptomycin B (Cell Signaling Technology). Buffer B was supplemented with 110 U of Protector RNAse Inhibitor and 8×10^−5^ M leptomycin B. After the final spin in Buffer A, the nuclei were fixed in Riley's Buffer [PBS supplemented with 1% bovine serum albumin (BSA), 146 mM NaCl, 10 mM Tris-HCl, 1 mM CaCl_2_, 21 mM MgCl_2_, 20 U of SUPERaseIn (Invitrogen) and 8×10^−5^ M leptomycin B] supplemented with 0.1% PFA. After fixation, the nuclei were centrifuged at 500 ***g*** for 4 min, resuspended in Riley's Buffer without PFA, and centrifuged again at 500 ***g*** for 4 min. After centrifuging the nuclei in Buffer B as directed by the manufacturer's protocol, the nuclei were resuspended in Nuclear Storage Buffer (PBS supplemented with 1% BSA, 200 U of Protector RNAse Inhibitor and 8×10^−5^ M leptomycin B). An aliquot of the nuclei was stained with Trypan Blue, and nuclear concentration was determined using a TC20 Automated Cell Counter. Nuclei were assessed for intactness using a Nikon Eclipse Ti prior to submission to the Single Cell Genomics Core at Cincinnati Children's Hospital Medical Center for 10x Genomics sequencing. One graft was sequenced per time point.

### Single-cell extractions

The methodology used has been described in detail by McCauley et al. (2023), as data from these grafts were previously published. Briefly, tHIOs were collected in ice-cold PBS, cut into small pieces, and cleared of mucus. Organoids were incubated in TrypLE Select (Gibco) with 10 μM Y-27632 ROCK inhibitor (Tocris) for 60-90 min at 4°C with vigorous shaking every 10 min. Samples were spun down at 500 ***g*** at 4°C, resuspended in 10% fetal bovine serum in DMEM, and filtered through BSA-precoated 100 µm filter. Remaining undissociated tissue pieces were incubated in Accutase for 10 min at 37°C. All cells were washed, resuspended in PBS with 0.5% BSA, 5% fetal bovine serum and 10 µM Y-27632 and passed through a 40 µM filter. Sytox Blue (Thermo Fisher Scientific)-negative viable cells were enriched using fluorescence-activated cell sorting on a BD FACS Aria II. Viable cells were processed for scRNA-seq. Approximately 12,800 cells were loaded with an estimated capture of 8000 cells per sample.

### Single cell and nucleus library preparation

All snRNA-seq and scRNA-seq libraries were prepared using the 10x Genomics Chromium platform using version 3.1 chemistry. Sequencing was performed using a NovaSeq 6000 (Illumina) machine.

### Single cell and nucleus data processing

Cell Ranger v3.02.2 (10x Genomics) was used to align the snRNA and scRNA FASTQ files to the human hg38 genome, outputting a counts matrix while also generating quality control statistics (https://github.com/10XGenomics/cellranger). For the snRNA data, SoupX was used to adjust the counts by correcting for cell-free mRNA (Young and Behjati, 2020). This served as the input for the single-cell workflow in the R package Seurat (Stuart et al., 2019). For all samples, the counts were initially processed to remove cells with fewer than 100 genes or genes in fewer than three cells. ‘NormalizeData’ was used to perform per-cell normalization and log-transform the data, and ‘FindVariableFeatures’ was used to identify the top 2000 genes with the highest cell-to-cell variation. The ‘ScaleData’ function was used to transform the counts to have mean 0 and variance 1, which is a necessary step for ‘RunPCA’, which was used to perform a linear dimensionality reduction method called principal component analysis (PCA). The first 20 principal components were used for the functions ‘FindNeighbors’ and ‘FindClusters’, to generate a graph and identify clusters of cells using k-nearest neighbors. Finally, ‘RunUMAP’ was used to perform a second, non-linear dimensionality reduction on the principal components called uniform manifold approximation projection (UMAP), which was used for generating figures.

### Single-nucleus sample integration

To construct a combined atlas of the snRNA datasets we performed integration as implemented in Seurat. The ‘FindIntegrationAnchors’ function takes a list of individual Seurat objects and uses canonical correlation analysis (CCA) on them for dimensionality reduction. The resulting canonical correlation vectors were normalized and mutual nearest neighbors (MNNs) were found in the low dimensional space. The ‘IntegrateData’ uses these neighbors or ‘anchors’ to calculate a correction vector, which in turn was used to remove batch effects from the joint expression matrix. Finally, the integrated dataset was reprocessed (RunPCA, FindNeighbors, FindClusters and RunUMAP) and visualized.

### Constructing a single-cell reference atlas for annotation

snRNA samples were annotated using a supervised learning approach that requires a reference dataset. To take advantage of different cell types across studies, a reference atlas was created from three fetal gut datasets (E-MTAB-8901, GSE158702 and E-MTAB-10187) using ELeFHAnt (https://github.com/praneet1988/ELeFHAnt). Each dataset was initially filtered to include only intestinal-derived cells and cell types of interest. ELeFHAnt's ‘LabelHarmonization’ function was then used to perform Seurat's CCA integration to combine the datasets while removing batch effects. The atlas at this point contained a total of 70 cell types across 62 clusters and ∼115,000 cells. To resolve conflicting labels, the atlas was split into training and test sets, and the optimal cell type for each cluster was learned using multiple classifiers. The final output of ‘LabelHarmonization’ was the integrated atlas with 41 harmonized cell types. Further modifications to the cell-type names were made through manual curation.

### Annotation of snRNA-seq and scRNA-seq samples

Following construction of the harmonized reference atlas using ELeFHAnt, the same methods were used to annotate the snRNA data. The package implements an ensemble-supervised, machine-learning approach whereby multiple classifiers, including random forest and support vector machine train, predict cell types for new datasets by learning from reference datasets. Prior to training, the ‘ClassifyCells’ function downsamples the reference and selects the top 2000 variable features shared between reference and query. Cell types are then predicted for each cell using the ensemble-learning method.

Alternatively, Seurat provides a method of annotation through the ‘MapQuery’ function. The reference atlas PCA is projected onto each query dataset, and transcriptionally similar pairs of cells are found between them. These ‘anchors’ are used by a weighted vote classifier to find the optimal reference cell type for each query cell.

### scRNA versus snRNA comparison

scRNA datasets of tHIOs were compared with the snRNA dataset of an 8 week tHIO as follows. First, the number of cells and genes measured for each dataset were found. Dot plots displaying the read versus gene counts on a log10 scale for each cell were produced. All datasets were then combined using Seurat's CCA integration to visualize how closely different datasets group together in the UMAP. The scRNA and snRNA datasets were integrated separately as well, and MapQuery was used to project cell types from one to the other as well as onto the harmonized reference atlas.

### Pseudotime trajectory analysis using VIA

To investigate cell differentiation over time, trajectory analysis was performed on the integrated single nuclear and single cell atlases using VIA (Stassen et al., 2021). To prepare the data, each atlas was divided into mesenchymal and epithelial subsets and converted to the Anndata format. PCA and UMAP were also performed on each dataset using SCANPY (Wolf et al., 2018). Finally, the VIA function was run using the default parameters and specifying the progenitor or root cell types for each dataset. The graph-based method determines pseudotime using lazy-teleporting random walks and Monte-Carlo Markov chains before predicting terminal states and lineages.

The analysis was performed in two iterations with different granularity levels to produce two variations of the trajectory. For visualization, pseudotime values were extracted and displayed on each dataset's UMAP using Seurat.

### Delineating pseudotime and differentiation potential within lineages

Cells from lineages of interest (LGR5^+^ stem cells and telocytes) were extracted from each of the three single-cell atlases that comprised the reference atlas using the ‘Subset’ function from Seurat (R package v4). Cells from three atlases were integrated using a CCA algorithm, which intrinsically uses an MNN-based approach to integrate cells. Integrated cells were then used supplied to ‘Slingshot’ (trajectory inference R package v2.5.2) to obtain trajectory (pseudo time) followed by running ‘FitGam’ (fit additive models for gene expression smoothening). FitGam expression was then tested using differential tests (‘associationTest’, ‘startVsEndTest’, ‘diffEndTest’) to identify genes changing across, at the start, and at the end of the pseudo time. Expression changes were plotted using a heatmap. We further tested the change of gene expression across time points using CytoTRACE (R package; https://cytotrace.stanford.edu/), which checks for the differentiation potential of cells based on number of genes expressed. The ‘iCytoTRACE’ function was applied to the count matrix of cells of interest from the three atlases to correct for expression using MNNs and identify differentiation potential of cells.

### Receptor-ligand analysis

Seurat's ‘FeaturePlot’ function was employed to visualize expression of key pathway genes from WNT, HIPPO/YAP and NRG1 pathways. min.cutoff =‘q10’ and order=TRUE settings were applied to improve the contrast between cells.

## Supplementary Material

10.1242/develop.201416_sup1 Supplementary information
