# Supplementary material for: Transplanted human intestinal organoids: a resource for modeling human intestinal development
Source: Development. 2023 May 5;150(9):dev201416. doi: 10.1242/dev.201416 (PMC10259511; doi:10.1242/dev.201416)
Supplement: Supplementary information [file develop-150-201416-s1.pdf]

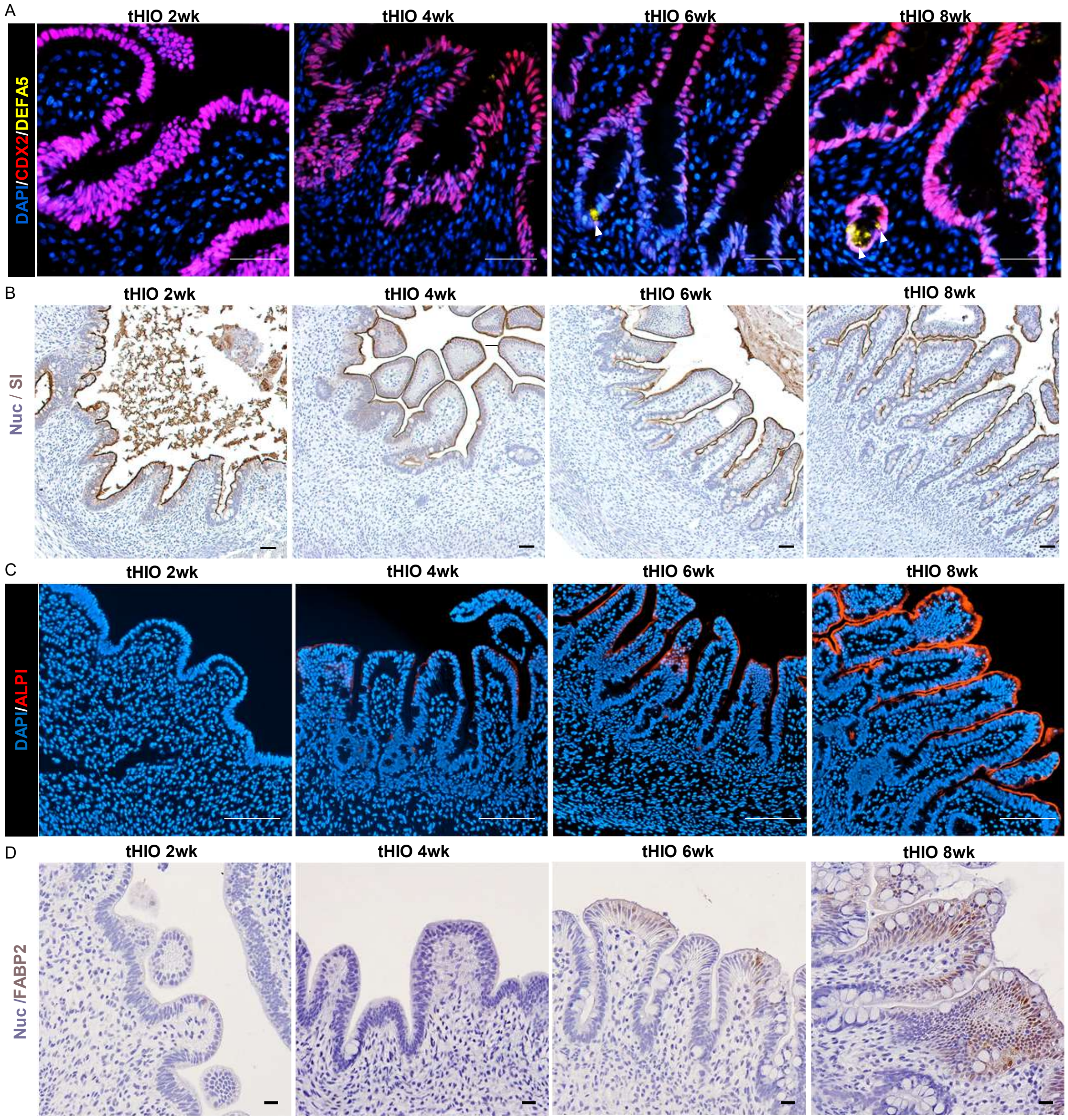

Fig. S1. The tHIO system can be used to study cellular maturation over time (n=5 per timepoint)**.** (A) Staining for DEFA5 (yellow), a marker of Paneth cells (white arrowhead), and CDX2 (red), a marker of intestinal epithelium, across the tHIO timecourse. (B) Staining for SI (brown), a brush border enzyme that marks enterocytes. (C) Staining for ALPI (red), a marker of enterocyte function, across the tHIO timecourse. (D) Staining for FABP2 (brown), a fatty acid transporter, over time.

Development • Supplementary information

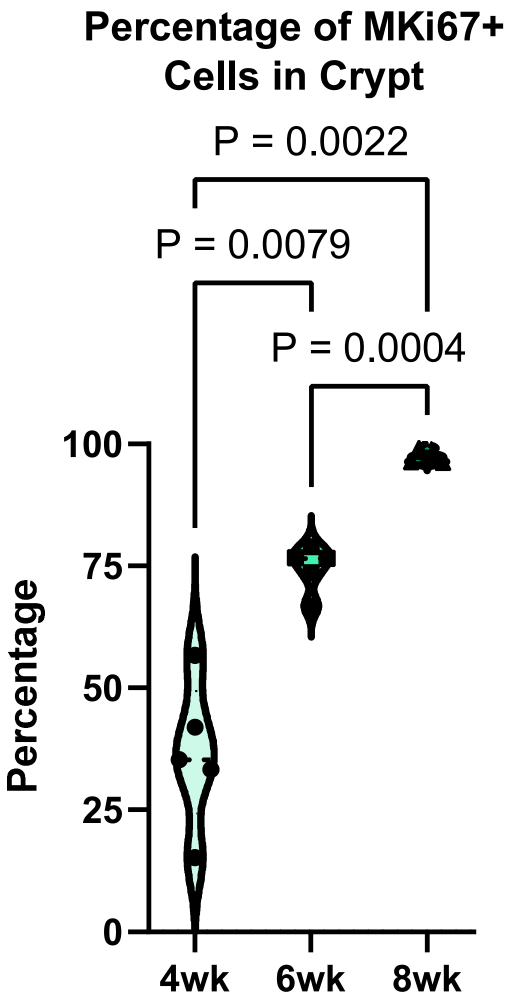

Fig. S2. Over time, crypt cells MKi67+ cells become restricted to the intestinal crypts in tHIOs. (n=5 per timepoint). P-values were assigned using a Brown-Forsythe and Welch ANOVA one-tailed test. A Dunnett T3 test was performed to correct for multiple comparisons.

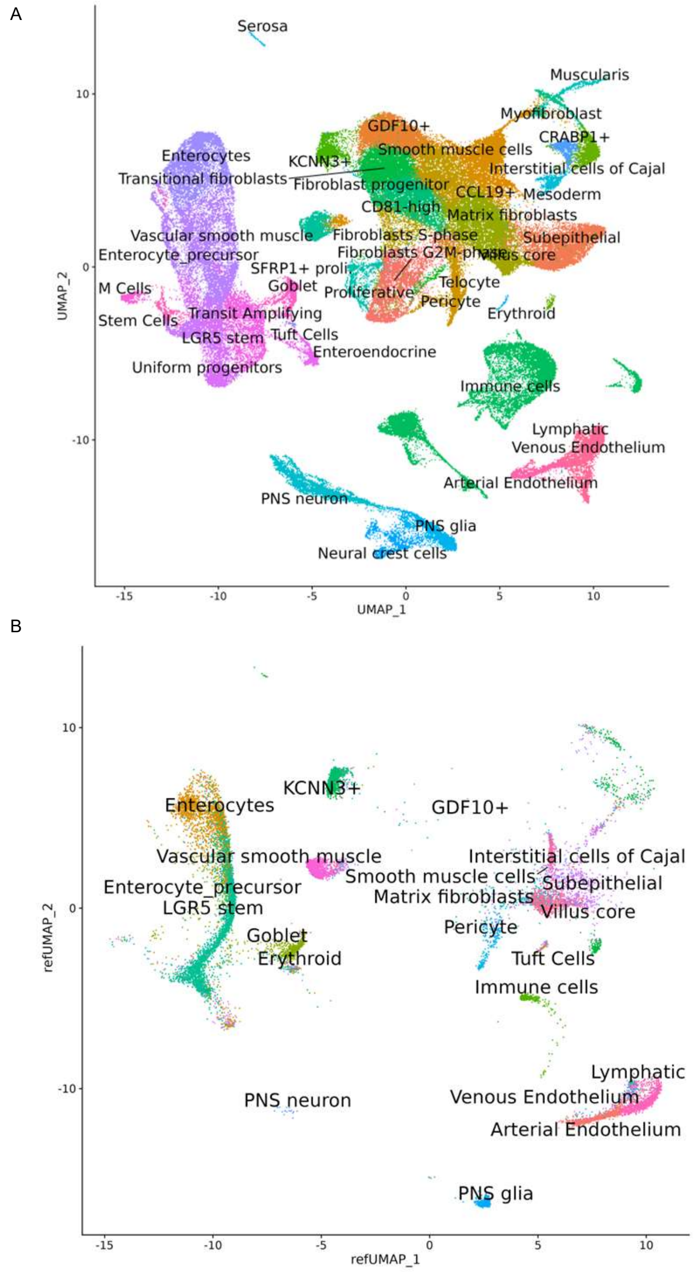

Fig. S3. Single cell analysis of patient human intestine. (A) UMAP of Reference Atlas constructed from three publicly available single cell RNA sequencing datasets. Reference Atlas was used to label tHIO samples and patient human intestine sample. (B) Single nucleus RNA sequencing dataset generated from a full-thickness patient sample (n=1). Single nucleus extraction protocol successfully isolated nuclei from a diverse range of cell types, including epithelial and mesenchymal cells.

Development • Supplementary information

A

Upregulated Early Biological Processes: LGR5+ Cells in the Reference Atlas

GO Type	Number	Process
GO: Biological Process	GO:0006412	translation
GO: Biological Process	GO:0043043	peptide biosynthetic process
GO: Biological Process	GO:0006518	peptide metabolic process
GO: Biological Process	GO:0002181	cytoplasmic translation
GO: Biological Process	GO:0043604	amide biosynthetic process
GO: Biological Process	GO:0043603	cellular amide metabolic process
GO: Biological Process	GO:0034645	cellular macromolecule biosynthetic process
GO: Biological Process	GO:0032543	mitochondrial translation
GO: Biological Process	GO:0140053	mitochondrial gene expression
GO: Biological Process	GO:0042254	ribosome biogenesis

Upregulated Late Biological Processes: LGR5+ Cells in the Reference Atlas

GO Type	Number	Process
GO: Biological Process	GO:0006412	translation
GO: Biological Process	GO:0043043	peptide biosynthetic process
GO: Biological Process	GO:0043604	amide biosynthetic process
GO: Biological Process	GO:0006518	peptide metabolic process
GO: Biological Process	GO:0002181	cytoplasmic translation
GO: Biological Process	GO:0043603	cellular amide metabolic process
GO: Biological Process	GO:0032543	mitochondrial translation
GO: Biological Process	GO:0034645	cellular macromolecule biosynthetic process
GO: Biological Process	GO:0140053	mitochondrial gene expression
GO: Biological Process	GO:0022613	ribonucleoprotein complex biogenesis

B

Upregulated Early Biological Processes: LGR5+ Cells in the tHIOs

GO Type	Number	Process
GO: Biological Process	GO:0048666	neuron development
GO: Biological Process	GO:0031175	neuron projection development
GO: Biological Process	GO:0034330	cell junction organization
GO: Biological Process	GO:0000902	cell morphogenesis
GO: Biological Process	GO:0050808	synapse organization
GO: Biological Process	GO:0000904	cell morphogenesis involved in differentiation
GO: Biological Process	GO:0072359	circulatory system development
GO: Biological Process	GO:0120039	plasma membrane bounded cell projection morphogenesis
GO: Biological Process	GO:0060429	epithelium development
GO: Biological Process	GO:0048858	cell projection morphogenesis

Upregulated Late Biological Processes: LGR5+ Cells in the tHIOs

GO Type	Number	Process
GO: Biological Process	GO:0000902	cell morphogenesis
GO: Biological Process	GO:0007010	cytoskeleton organization
GO: Biological Process	GO:0032880	regulation of protein localization
GO: Biological Process	GO:0060341	regulation of cellular localization
GO: Biological Process	GO:0030029	actin filament-based process
GO: Biological Process	GO:0046907	intracellular transport
GO: Biological Process	GO:0033043	regulation of organelle organization
GO: Biological Process	GO:0044265	cellular macromolecule catabolic process
GO: Biological Process	GO:0022603	regulation of anatomical structure morphogenesis
GO: Biological Process	GO:0048858	cell projection morphogenesis

C

Upregulated Early Biological Processes: Telocytes

GO Type	Number	Process
GO: Biological Process	GO:0061061	muscle structure development
GO: Biological Process	GO:0072359	circulatory system development
GO: Biological Process	GO:0007155	cell adhesion
GO: Biological Process	GO:0009887	animal organ morphogenesis
GO: Biological Process	GO:0009611	response to wounding
GO: Biological Process	GO:0035295	tube development
GO: Biological Process	GO:0009725	response to hormone
GO: Biological Process	GO:0060429	epithelium development
GO: Biological Process	GO:0048598	embryonic morphogenesis
GO: Biological Process	GO:0035239	tube morphogenesis

Upregulated Late Biological Processes: Telocytes

GO Type	Number	Process
GO: Biological Process	GO:0007155	cell adhesion
GO: Biological Process	GO:0040011	locomotion
GO: Biological Process	GO:0048646	anatomical structure formation involved in morphogenesis
GO: Biological Process	GO:0009887	animal organ morphogenesis
GO: Biological Process	GO:0072359	circulatory system development
GO: Biological Process	GO:0040012	regulation of locomotion
GO: Biological Process	GO:0035295	tube development
GO: Biological Process	GO:0043067	regulation of programmed cell death
GO: Biological Process	GO:0001944	vasculature development
GO: Biological Process	GO:0060429	epithelium development

D

Upregulated Early Biological Processes: Telocytes

GO Type	Number	Process
GO: Biological Process	GO:0031175	neuron projection development
GO: Biological Process	GO:0048666	neuron development
GO: Biological Process	GO:0000902	cell morphogenesis
GO: Biological Process	GO:0030334	regulation of cell migration
GO: Biological Process	GO:0061061	muscle structure development
GO: Biological Process	GO:0072359	circulatory system development
GO: Biological Process	GO:0048729	tissue morphogenesis
GO: Biological Process	GO:0060485	mesenchyme development
GO: Biological Process	GO:0035239	tube morphogenesis
GO: Biological Process	GO:0060429	epithelium development

Upregulated Late Biological Processes: Telocytes

GO Type	Number	Process
GO: Biological Process	GO:0048666	neuron development
GO: Biological Process	GO:0000902	cell morphogenesis
GO: Biological Process	GO:0048589	developmental growth
GO: Biological Process	GO:0072359	circulatory system development
GO: Biological Process	GO:0002064	epithelial cell development
GO: Biological Process	GO:0035295	tube development
GO: Biological Process	GO:0019827	stem cell population maintenance
GO: Biological Process	GO:0000904	cell morphogenesis involved in differentiation
GO: Biological Process	GO:0007507	heart development
GO: Biological Process	GO:0008593	regulation of Notch signaling pathway

Fig. S4. LGR5+ stem cells and telcoytes remain transcriptionally stable over time. (A) Upregulated early (left) and late (right) biological processes of the Reference Atlas. (B) Upregulated early (left) and late (right) biological processes of the tHIO timecourse. .(C) Upregulated early (left) and late (right) biological processes of the Reference Atlas. (D) Upregulated early (left) and late (right) biological processes of the tHIO timecourse.

Development • Supplementary information

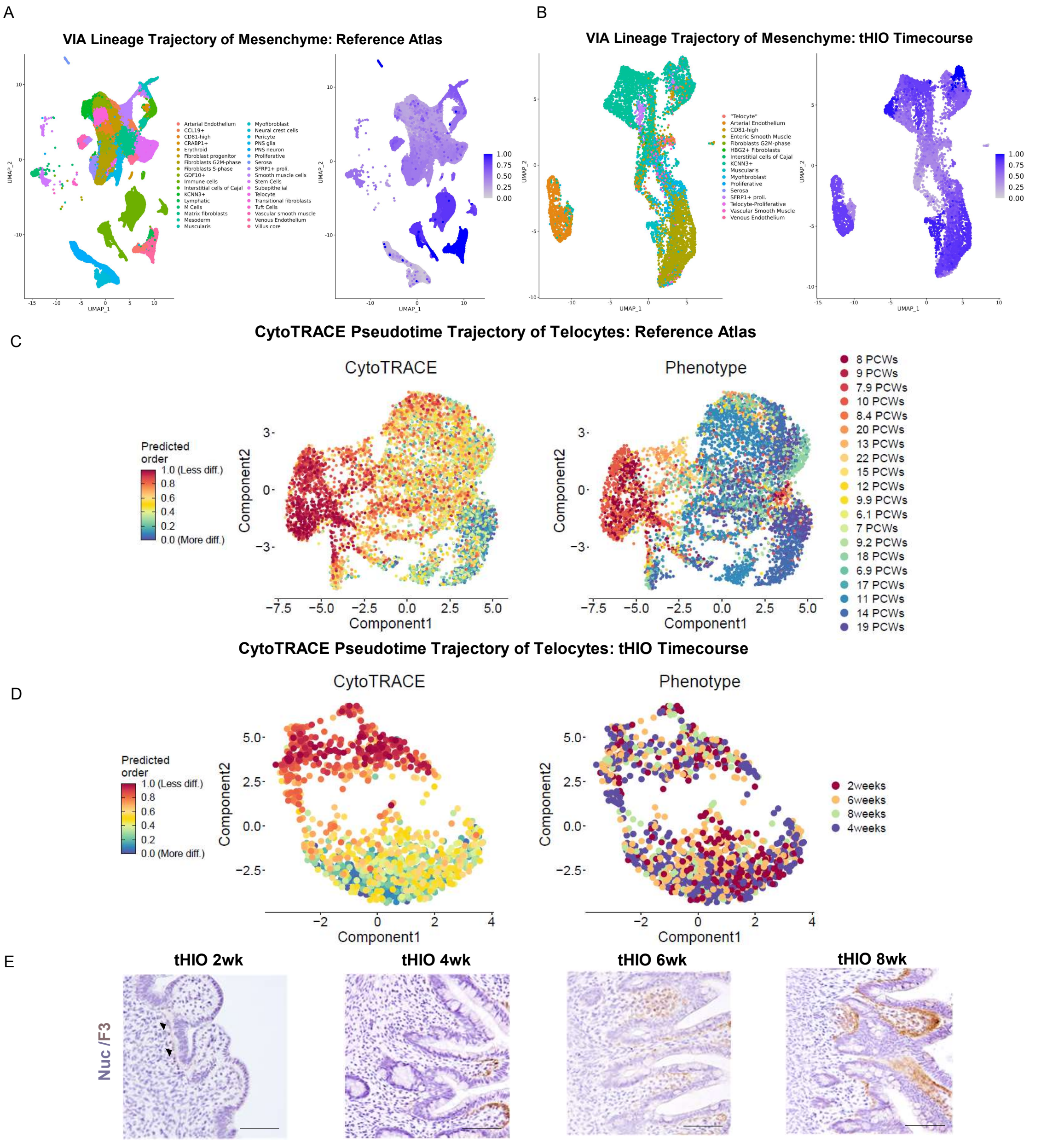

Fig. S5. Mesenchymal development in tHIOs mimics fetal human intestinal mesenchymal development (A) UMAP and VIA Pseudotime Trajectory of mesenchymal cell development in the Reference Atlas (B) UMAP and VIA Pseudotime Trajectory of mesenchymal cell development in the tHIO timecourse (C) CytoTRACE Pseudotime Trajectory of telocytes in the Reference Atlas (D) CytoTRACE Pseudotime Trajectory of telocytes in the tHIO timecourse. (E) Staining for F3 (brown), a marker of subepithelial cells, over time.

Development • Supplementary information

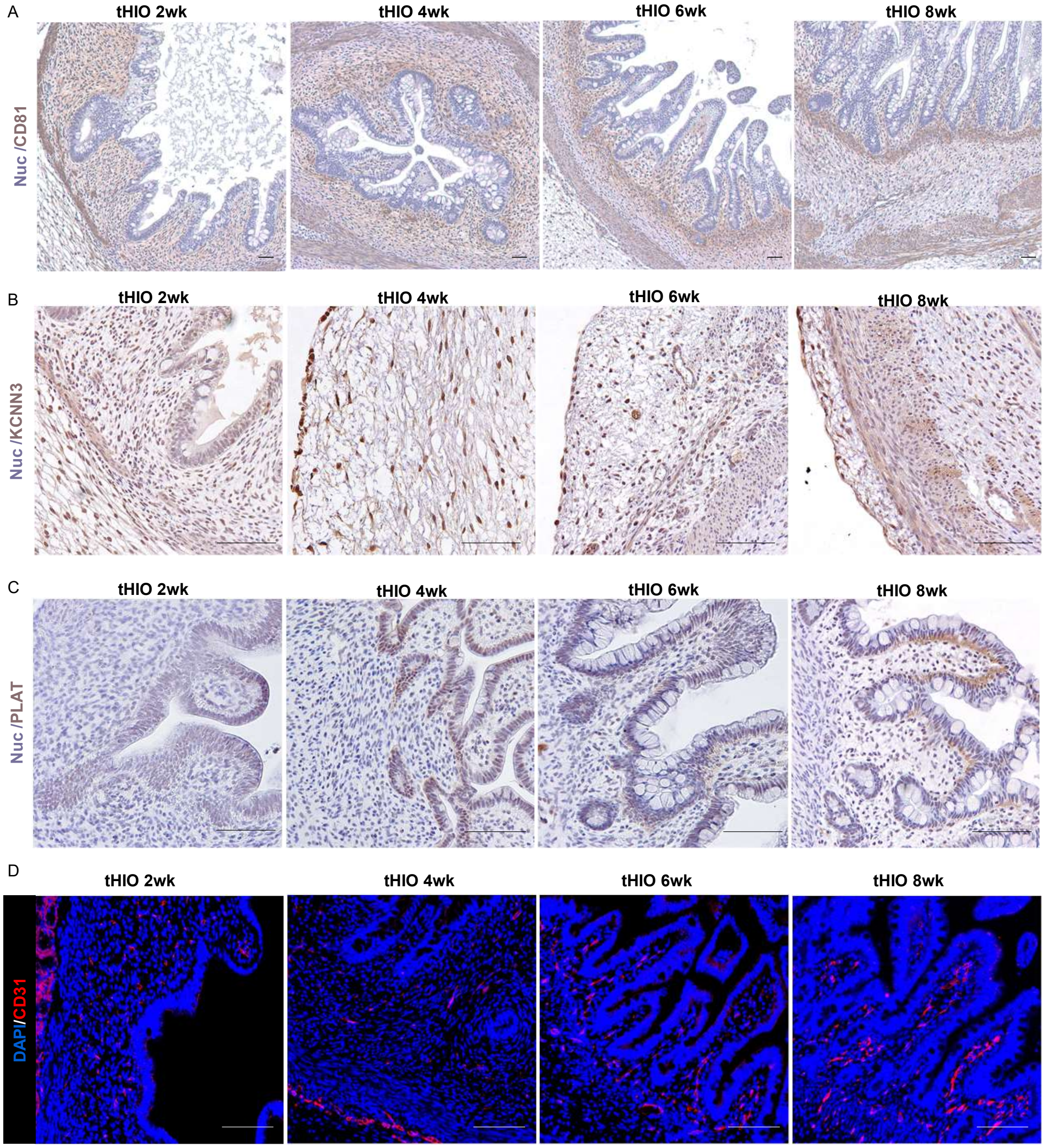

Fig. S6. The tHIO system can be used to study the maturation of the mesenchyme over time. (n=5 per timepoint) (A) Staining for CD81 (brown), a marker of CD81 high cells, across the tHIO timecourse. (B) Staining for KCCN3 (brown), a marker of KCNN3+ fibroblasts, across the tHIO timecourse. (C) Staining for PLAT (brown), a marker of myofibroblasts, across the tHIO timecourse. (D) Staining for CD31 (red), a marker of endothelial cells, over time.

A

Upregulated Biological Processes in Subepithelial Cells

GO TYPE	Number	Process
GO: Biological Process	GO:0042127	regulation of cell population proliferation
GO: Biological Process	GO:0051093	negative regulation of developmental process
GO: Biological Process	GO:0045595	regulation of cell differentiation
GO: Biological Process	GO:0035295	tube development
GO: Biological Process	GO:0045596	negative regulation of cell differentiation
GO: Biological Process	GO:0035239	tube morphogenesis
GO: Biological Process	GO:0030334	regulation of cell migration
GO: Biological Process	GO:0048585	negative regulation of response to stimulus
GO: Biological Process	GO:2000145	regulation of cell motility
GO: Biological Process	GO:0040012	regulation of locomotion

Downregulated Biological Processes in Subepithelial Cells

GO TYPE	Number	Process
GO: Biological Process	GO:0048285	organelle fission
GO: Biological Process	GO:0000280	nuclear division
GO: Biological Process	GO:0000278	mitotic cell cycle
GO: Biological Process	GO:0140014	mitotic nuclear division
GO: Biological Process	GO:1903047	mitotic cell cycle process
GO: Biological Process	GO:0051301	cell division
GO: Biological Process	GO:0022402	cell cycle process
GO: Biological Process	GO:0051726	regulation of cell cycle
GO: Biological Process	GO:0044772	mitotic cell cycle phase transition
GO: Biological Process	GO:0044770	cell cycle phase transition

B

Upregulated Biological Processes in Myofibroblasts

GO TYPE	Number	Process
GO: Biological Process	GO:0007167	enzyme linked receptor protein signaling pathway
GO: Biological Process	GO:0006613	cotranslational protein targeting to membrane
GO: Biological Process	GO:0050673	epithelial cell proliferation
GO: Biological Process	GO:0045596	negative regulation of cell differentiation
GO: Biological Process	GO:0045668	negative regulation of osteoblast differentiation
GO: Biological Process	GO:0009887	animal organ morphogenesis
GO: Biological Process	GO:0030278	regulation of ossification
GO: Biological Process	GO:0031099	regeneration
GO: Biological Process	GO:0050679	positive regulation of epithelial cell proliferation
GO: Biological Process	GO:0001568	blood vessel development

Downregulated Biological Processes in Myofibroblasts

GO TYPE	Number	Process
GO: Biological Process	GO:0001501	skeletal system development
GO: Biological Process	GO:0030198	extracellular matrix organization
GO: Biological Process	GO:0043062	extracellular structure organization
GO: Biological Process	GO:0045229	external encapsulating structure organization
GO: Biological Process	GO:0051241	negative regulation of multicellular organismal process
GO: Biological Process	GO:0060425	lung morphogenesis
GO: Biological Process	GO:0050650	chondroitin sulfate proteoglycan biosynthetic process
GO: Biological Process	GO:0061138	morphogenesis of a branching epithelium
GO: Biological Process	GO:0001763	morphogenesis of a branching structure
GO: Biological Process	GO:0002009	morphogenesis of an epithelium

C

Upregulated Biological Processes in CD81 High Cells

GO TYPE	Number	Process
GO: Biological Process	GO:0043062	extracellular structure organization
GO: Biological Process	GO:0045229	external encapsulating structure organization
GO: Biological Process	GO:0030199	collagen fibril organization
GO: Biological Process	GO:0001501	skeletal system development
GO: Biological Process	GO:0001503	ossification
GO: Biological Process	GO:0009887	animal organ morphogenesis
GO: Biological Process	GO:0048286	lung alveolus development
GO: Biological Process	GO:0009719	response to endogenous stimulus
GO: Biological Process	GO:2000647	negative regulation of stem cell proliferation
GO: Biological Process	GO:0071230	cellular response to amino acid stimulus
GO: Biological Process	GO:0045595	regulation of cell differentiation

Downregulated Biological Processes in CD81 High Cells

GO TYPE	Number	Process
GO: Biological Process	GO:0051301	cell division
GO: Biological Process	GO:0000280	nuclear division
GO: Biological Process	GO:0051726	regulation of cell cycle
GO: Biological Process	GO:0048285	organelle fission
GO: Biological Process	GO:0022402	cell cycle process
GO: Biological Process	GO:0000278	mitotic cell cycle
GO: Biological Process	GO:0140014	mitotic nuclear division
GO: Biological Process	GO:1903047	mitotic cell cycle process
GO: Biological Process	GO:0000819	sister chromatid segregation
GO: Biological Process	GO:0000070	mitotic sister chromatid segregation

D

Upregulated Biological Processes in KCNN3+ Fibroblasts

GO TYPE	Number	Process
GO: Biological Process	GO:0030198	extracellular matrix organization
GO: Biological Process	GO:0043062	extracellular structure organization
GO: Biological Process	GO:0045229	external encapsulating structure organization
GO: Biological Process	GO:0070848	response to growth factor
GO: Biological Process	GO:0009887	animal organ morphogenesis
GO: Biological Process	GO:0042340	keratan sulfate catabolic process
GO: Biological Process	GO:0007155	cell adhesion
GO: Biological Process	GO:0022610	biological adhesion
GO: Biological Process	GO:0006936	muscle contraction
GO: Biological Process	GO:0044273	sulfur compound catabolic process

Downregulated Biological Processes in KCNN3+ Fibroblasts

GO TYPE	Number	Process
GO: Biological Process	GO:0051301	cell division
GO: Biological Process	GO:0060485	mesenchyme development
GO: Biological Process	GO:0016477	cell migration
GO: Biological Process	GO:0009790	embryo development
GO: Biological Process	GO:0050673	epithelial cell proliferation
GO: Biological Process	GO:0048568	embryonic organ development
GO: Biological Process	GO:0045595	regulation of cell differentiation
GO: Biological Process	GO:0045596	negative regulation of cell differentiation
GO: Biological Process	GO:0048762	mesenchymal cell differentiation
GO: Biological Process	GO:0030334	regulation of cell migration

Fig. S7. Functions of select mesenchymal cell types. (A) Upregulated (left) and downregulated (right) GO terms for subepithelial cells. (B) Upregulated (left) and downregulated (right) GO terms for myofibroblasts. (C) Upregulated (left) and downregulated (right) GO terms for CD81 high cells. (D) Upregulated (left) and downregulated (right) GO terms for KCNN3+ fibroblasts.

Development • Supplementary information

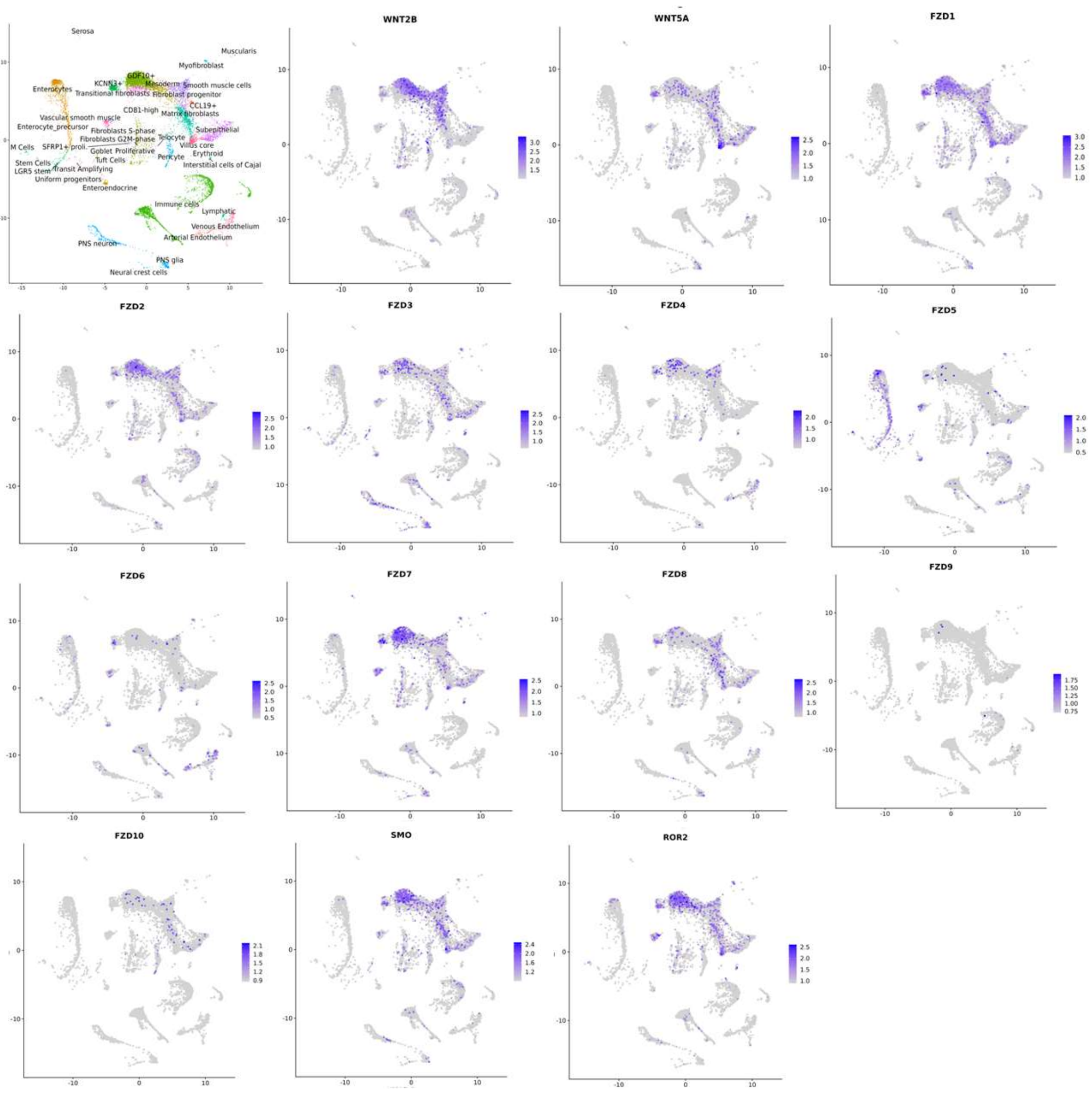

Fig. S8. Receptor-Ligand analysis of key niche factors Wnt2b and Wnt5a in GW18 human intestine.

Development • Supplementary information

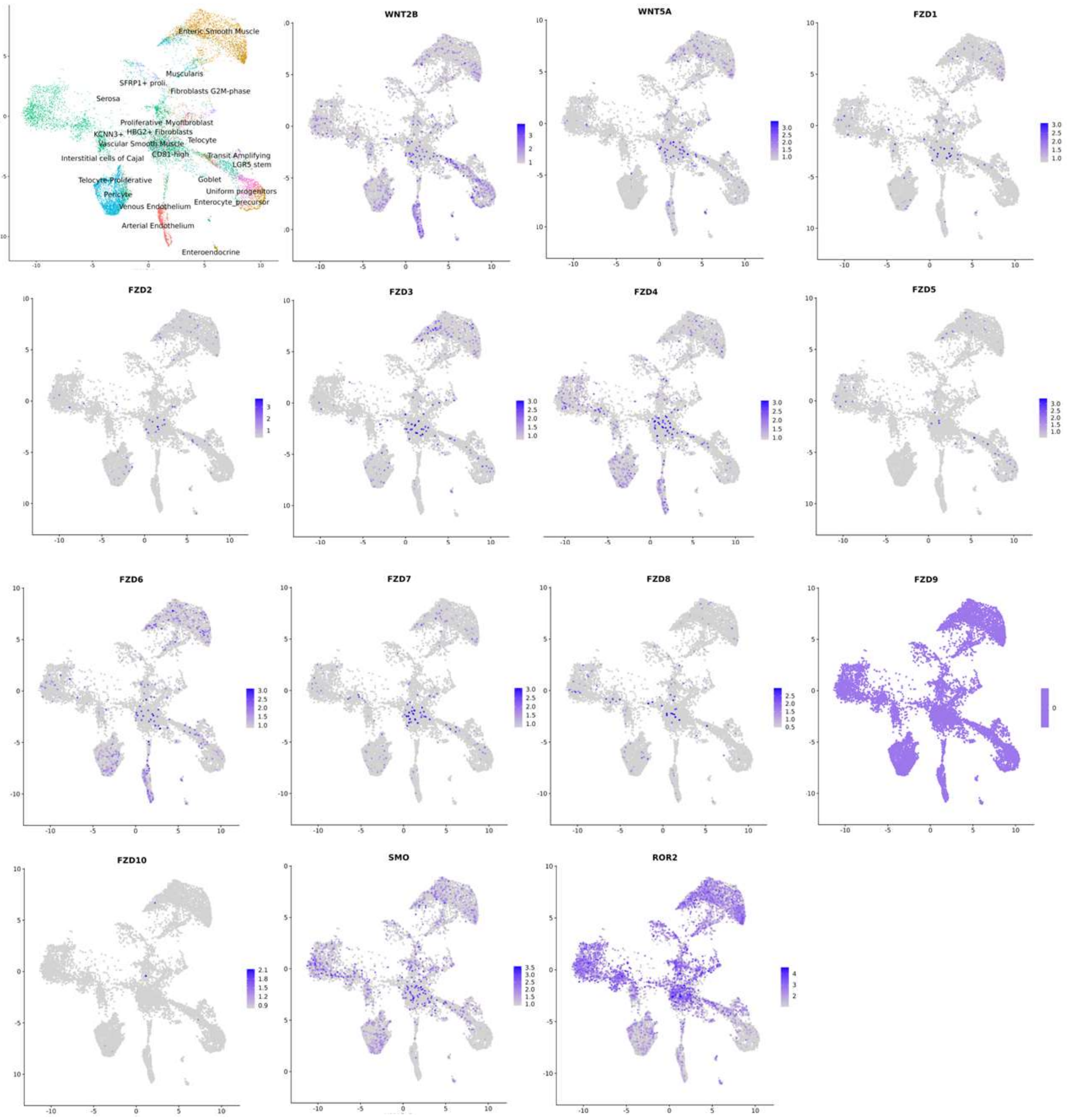

Fig. S9. Receptor-Ligand analysis of key niche factors Wnt2b and Wnt5a in 8wk tHIO.

Development • Supplementary information

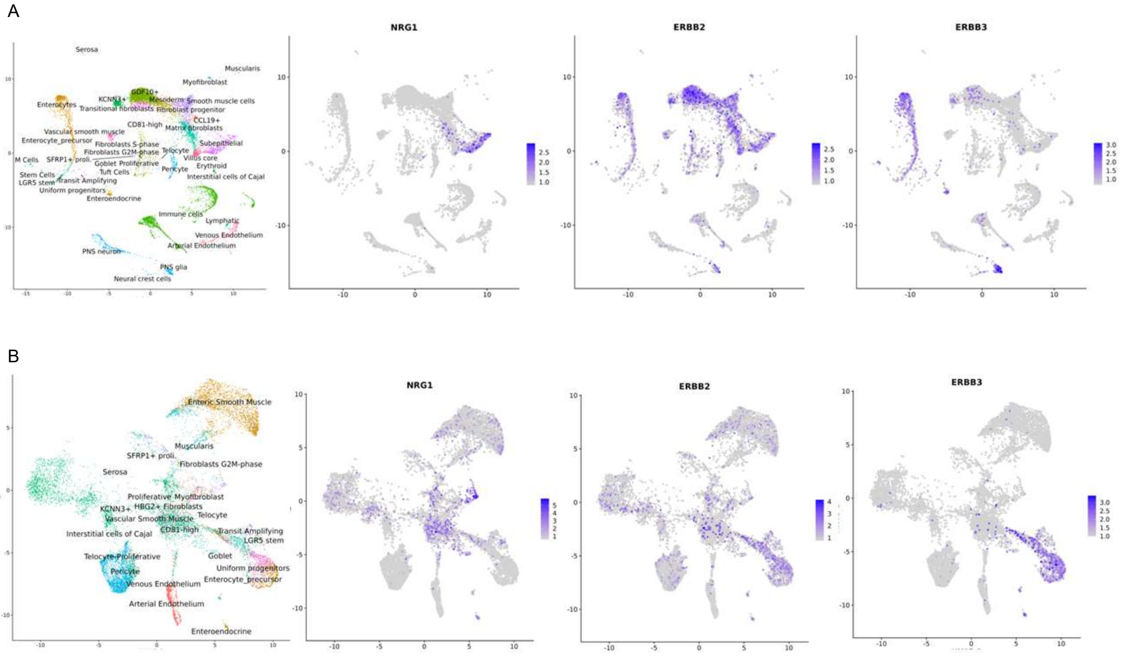

Fig. S10. Receptor-Ligand analysis of key niche factor NRG1 in GW 18 human intestine (A) and 8wk tHIO (B).

Development • Supplementary information

Development: doi:10.1242/dev.201416 Supplementary information

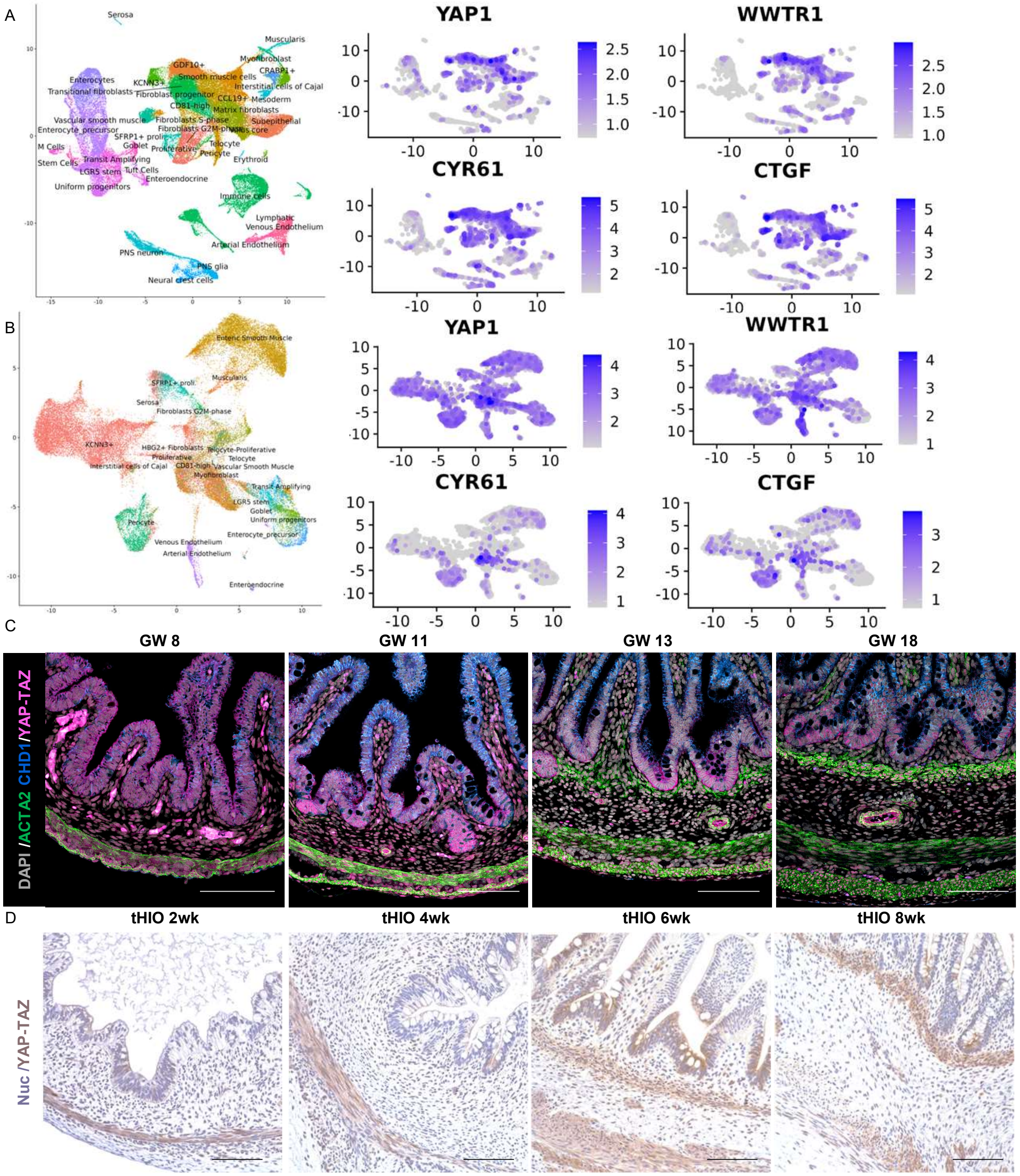

Fig. S11. The HIPPO/YAP pathway plays a role in regulating enteric smooth muscle development. (A) Feature plots showing transcription of YAP1, WWTR1, and YAP target genes CYR61 and CTGF in the mesenchyme of GW18 human fetal intestine. (B) Feature plots showing transcription of YAP1, WWTR1, and YAP target genes CYR61 and CTGF in the mesenchyme of 8wk tHIO. (C) Staining for ACTA2 (green), CDH1 (blue) and the YAP-TAZ complex (pink) at key timepoints in fetal human intestine (n=1 per timepoint). (D) Staining for the YAP-TAZ complex (brown) across the tHIO timecourse (n=5 per timepoint).

Table S1. Antibody Information for Immunohistochemistry and Immunofluorescence Staining.

	Antigen	Dilution	Host	Company: Catalog Number
Primary	Anillin (ANLN)	1:100	Rabbit	Atlas: HPA050556
	Platelet Endothelial Cell Adhesion Molecule (CD31)	1:100	Rabbit	Abcam: ab28364
	Tetraspanin-28 (CD81)	1:250	Rabbit	Atlas: HPA007234
	Chromogranin A (CHGA)	1:500	Mouse	DSHB: CPTC-CHGA-1
	Defensin Alpha 5 (DEFA5)	1:500	Mouse	Abcam: ab90802
	E-Cadherin (CDH1)	1:300	Mouse	BD: 610182
	Elastin Microfibril Interfacer 1 (EMILIN1)	1:400	Rabbit	Atlas: HPA002822
	Fatty Acid Binding Protein 2 (FABP2)	1:600	Rabbit	Atlas: HPA034607
	Coagulation Factor III (F3)	1:250	Rabbit	Atlas: HPA049292
	Potassium Calcium-Activated Channel Subfamily N Member 3 (KCNN3)	1:300	Rabbit	Atlas: HPA057127
	Leimodin 1 (LMOD1)	1:50	Rabbit	Atlas: HPA030097
	Lysozyme (hLYZ)	1:2000	Rabbit	Biorad: 0100-0523
	Marker Of Proliferation Ki-67 (MKI67)	1:350	Rabbit	Thermo Fisher: RM-9106-50
	Mucin-2 (MUC2)	1:1100	Rabbit	Abcam: ab134119
	Olfactomedin 4 (OLFM4)	1:200	Mouse	Cell Signalling: 14369
	Tissue Plasminogen Activator (PLAT)	1:100	Rabbit	Atlas: HPA003412
	Sucrase-Isomaltase (SI)	1:800	Rabbit	Sigma: HPA011897
	Vimentin (VIM)	1:350	Goat	Santa-Cruz: SC-7557
	α-goat AF647	1:800	Donkey	Life Technologies:A21447
	α-mouse AF568	1: 800	Donkey	Life Technologies: A10037
Secondary	α-rabbit AF647	1: 800	Donkey	Life Technologies: A31573
	α-rabbit AF488	1: 800	Donkey	Life Technologies: A21206
	α-rabbit Biotin	1:1000	Goat	Vector: BA-1000
	SA Cy3	1:500		Abcam: ab175704

Development • Supplementary information
