# Supplementary material for: Transplanted human intestinal organoids: a resource for modeling human intestinal development
Source: Development. 2023 May 5;150(9):dev201416. doi: 10.1242/dev.201416 (PMC10259511; doi:10.1242/dev.201416)
Supplement: Supplementary file 1 [file develop-150-201416_review_history.pdf]

Transplanted human intestinal organoids: a resource for modeling human intestinal development

Akaljot Singh, Holly M. Poling, Praneet Chaturvedi, Konrad Thorner, Nambirajan Sundaram, Daniel O. Kechele, Charlie J. Childs, Heather A. McCauley, Garrett W. Fisher, Nicole E. Brown, Jason R. Spence, James M. Wells and Michael Helmrath
DOI: 10.1242/dev.201416

Editor: Matthias Lutolf

Review timeline

Original submission:	4 November 2022
Editorial decision:	7 December 2022
First revision received:	13 March 2023
Accepted:	28 March 2023

Original submission

First decision letter

MS ID#: DEVELOP/2022/201416

MS TITLE: Transplanted Human Intestinal Organoids: A Resource for Modeling Human Intestinal Development

AUTHORS: Akaljot Singh, Holly M Poling, Praneet Chaturvedi, Konrad Thorner, Nambirajan Sundaram, Daniel O. Kechele, Charlie J. Childs, Heather A. McCauley, Garrett W. Fisher, Nicole E Brown, Jason R. Spence, James M Wells, and Michael Helmrath

I have now received all the referees' reports on the above manuscript, and have reached a decision. The referees' comments are appended below, or you can access them online: please go to BenchPress and click on the 'Manuscripts with Decisions' queue in the Author Area.

As you will see, the referees express considerable interest in your work, but have some significant criticisms and recommend a substantial revision of your manuscript before we can consider publication. If you are able to revise the manuscript along the lines suggested, which may involve further experiments, I will be happy receive a revised version of the manuscript. Your revised paper will be re-reviewed by one or more of the original referees, and acceptance of your manuscript will depend on your addressing satisfactorily the reviewers' major concerns. Please also note that Development will normally permit only one round of major revision. If it would be helpful, you are welcome to contact us to discuss your revision in greater detail. Please send us a point-by-point response indicating your plans for addressing the referee’s comments, and we will look over this and provide further guidance.

Please attend to all of the reviewers' comments and ensure that you clearly highlight all changes made in the revised manuscript. Please avoid using 'Tracked changes' in Word files as these are lost in PDF conversion. I should be grateful if you would also provide a point-by-point response detailing how you have dealt with the points raised by the reviewers in the 'Response to Reviewers' box. If you do not agree with any of their criticisms or suggestions please explain clearly why this is so.

© 2023. Published by The Company of Biologists under the terms of the Creative Commons Attribution License (https://creativecommons.org/licenses/by/4.0/).

Reviewer 1

Advance summary and potential significance to field

The authors build upon their previous body of work developing and investigating transplanted HIOs. This paper extends their findings by examining the time course of tHIO compared to atlases of human fetal development. The manuscript relied heavily upon mining existing datasets and using machine learning approaches to identify cell populations and map their developmental trajectories. Overall I have high enthusiasm for the findings and their relevance to intestinal research. As the authors point out, modeling early hyuman intestinal development has been challenging and the data they present helps to affirm tHIOs as a viable and useful model for early human development and disease processes. The manuscript is extremely well written, and the methodology section is very thorough and easy to follow and (at least conceptually) replicate. The findings in regard to mesenchymal development are a highlight of the technique and the manuscript and deserve emphasis.

Comments for the author

My suggestions for improving the manuscript are as follows:
1. The results and discussion section would benefit from a limitations section, particularly indicating the inherent limitations of their heavy reliance on machine learning and data extrapolation techniques for their fetal verification data.
2. It would be worth explaining in more detail while 8 weeks was selected as the terminal endpoint for the tHIOs.
3. Authors indicate that 5 grafts were harvested per timepoint, but it is unclear to this reader how many grafts were utilized for each single cell approach. Was only one graft used for snRNASeq and then 3 separate ones for scRNASeq for 3 replicates? Please clarify this.
4. Fig 1A. Might be more useful to write out the time points for the harvested tHIOs and align them with the human fetal times points rather than just generically saying comparison to human fetal time points. Maybe move that to the portion after the mouse picture and then put the analysis approaches at the end.
5. Fig. 1B I cannot read any of the labels, this needs to be edited or removed. if you zoomed into just one crypt you may be able to increase fonts. I also might move this to lower in the figure since you present actual data on the fetal components in 1C, and 1B could be a nice summary of the overlaps between the fetal and tHIOs timepoints if you moved it to the bottom.
6. Fig 1C and 1D. Can you indicate which muscle layer is being newly formed in each image since there are several highlighted in the figure?
7. Fig. 2C is it difficult to appreciate the olfm4 localization at this magnification, might be useful to use a higher magnification to highlight the crypts.
8. Fig 2D quantification of the Ki67 expression per region of the epithelium would be a useful adjunct.
9. figs 3C and 3C - difficult to read the labels at this font size. please increase size or add a legend.
10. Fig 4. I would like to see D and E enlarged to make it easier to appreciate the data. F and G could be move to a supplementary figure if needed. I would also add a header for F & G saying reference vs tHIO because you can't tell what you are looking at without referring to legend.
11. Supp Fig 1C - the Alpi staining appears to just be picking up the mucin layer, I am not seeing clear data that show it is staining enterocytes.
12. Supp Fig 2 - please increase label sizes or add a legend.
13. Supp Fig 3 - this is one of the most interesting components of the paper given the inherent difficulties in studying mesenchymal development and an advantage of this model system. I would consider moving it out of the supplement if space allows. Would also make space changes as suggested for the similar figure 4 as remarked in item 10 above.
Minor points:
page 9 line 1 - HIOs were transplanted page 23 bottom "Our findings suggest that tHIOs are a fantastic proxy for studying the development of the human fetal intestine." I would consider removing the word fantastic.

Reviewer 2

Advance summary and potential significance to field

© 2023. Published by The Company of Biologists under the terms of the Creative Commons Attribution License (https://creativecommons.org/licenses/by/4.0/).

For ethical and technical reasons, the spatiotemporal regulation of human intestinal development has been elusive. Singh et al. have carried out temporal single-cell transcriptomic analysis for transplanted human intestinal organoids and provided a valuable resource to understand human intestinal development. The authors employed transplanted human intestinal organoids, which recapitulate human intestinal development and thoroughly analyzed their transcriptomes at a single-cell level over time. Because this paper was submitted as a technical resource section, there are few novelties in the article.
However, the dataset is instructive for the community and will be suitable for Development if the authors address the following points.

Comments for the author

1. Although I understood this paper is in the resource paper format, the Results and Discussion sections are mainly descriptive. The authors should try to provide some analyses that could deepen the regulation of human intestinal development. For instance receptor-ligand analysis using scRNA/snRNAseq data would be helpful. Which mesenchymal subpopulation expresses important niche factors, such as Wnt2b?

2. Related to the above point, telocytes, one of the mesenchymal subpopulations, was reported to express Wnt niche factors for stem cell self-renewal. Yet, later reports have challenged this concept (McCarthy N et al. Cell Stem Cell 2020). Because these data are mainly derived from adult intestines, it would be interesting to delineate the Wnt and other niche factor expression profiles in the developmental mesenchymal subpopulations.

3. Page 17. The authors indicated “the emergence of intestinal function.” However, I am not sure this description is appropriate because they only provided functional marker expression data. “Function” implies intestinal digestion and absorption function, but simple gene/protein expression might be insufficient to conclude the biological function.

4. The authors showed poor efficient recovery of mesenchymal cells in their scRNA-seq analysis compared with snRNA-seq. However, the subpar recovery was simply due to the choice of digestive enzyme. According to their method sections, they used TrypLE Select to dissociate tHIOs for scRNAseq analysis. Because mesenchymal cells are embedded in ECM, including trypsin-resistant collagen, their method favors isolating epithelium but not mesenchyme. I would use trypsin for epithelial isolation and collagenase or other ECM degrader to isolate mesenchymal cells. It is probably unrealistic to redo the experiments and the authors should include this possibility in their paper.

5. Yap signal is activated in the mouse embryonic intestines. The authors focused on LGR5 stem cells, but it would be better to include YAP staining/YAP gene signature analysis during human intestinal development and tHIO development.

First revision

<u>Author response to reviewers' comments</u>

Reviewer 1 Advance Summary and Potential Significance to Field...

The authors build upon their previous body of work developing and investigating transplanted HIOs. This paper extends their findings by examining the time course of tHIO compared to atlases of human fetal development. The manuscript relied heavily upon mining existing datasets and using machine learning approaches to identify cell populations and map their developmental trajectories. Overall I have high enthusiasm for the findings and their relevance to intestinal research. As the authors point out, modeling early hyuman intestinal development has been challenging and the data they present helps to affirm tHIOs as a viable and useful model for early human development and disease processes. The manuscript is extremely well written, and the methodology section is very thorough and easy to follow and (at least conceptually) replicate. The findings in regard to

© 2023. Published by The Company of Biologists under the terms of the Creative Commons Attribution License (https://creativecommons.org/licenses/by/4.0/).

mesenchymal development are a highlight of the technique and the manuscript and deserve emphasis.

Reviewer 1 Comments for the Author...

My suggestions for improving the manuscript are as follows:

1. The results and discussion section would benefit from a limitations section, particularly indicating the inherent limitations of their heavy reliance on machine learning and data extrapolation techniques for their fetal verification data.

Thank you for this insight. We have added a discussion of the limitations of using a machine learning approach for cell type annotation to the Results & Discussion section.

2. It would be worth explaining in more detail while 8 weeks was selected as the terminal endpoint for the tHIOs.

8 weeks was selected as the endpoint because in the current kidney transplant model the grafts do not appear to mature further beyond this timepoint, both morphologically as well as from the development of specific epithelial cell types. We believe that the increased pressure from mucous and epithelial debri actually attenuate the villi. We have generated new models that allow for drainage and include ENS and Immune cells that do affect the graft and allow it to to taken to later time points, but these are beyond the kidney engraftment needed for early time points in this study. We have added these details to the "Materials and Methods" section of the manuscript.

3. Authors indicate that 5 grafts were harvested per timepoint, but it is unclear to this reader how many grafts were utilized for each single cell approach. Was only one graft used for snRNASeq and then 3 separate ones for scRNASeq for 3 replicates? Please clarify this.

For single nucleus RNA sequencing, one graft was used per timepoint. For single cell RNA sequencing, three grafts that were used. Some data from these grafts were recently published in McCauley *et al*, 2013. We have added these details to the "Materials and Methods" section of the manuscript.

4. Fig 1A. Might be more useful to write out the time points for the harvested tHIOs and align them with the human fetal times points rather than just generically saying comparison to human fetal time points. Maybe move that to the portion after the mouse picture and then put the analysis approaches at the end.

We added the specific tHIO and fetal human intestine timepoints to Figure 1A. To preserve clarity that single nucleus sequencing was performed on the tHIOs and not on fetal human intestine, we did not move the analysis portion of the image to the end.

5. Fig. 1B I cannot read any of the labels, this needs to be edited or removed. if you zoomed into just one crypt you may be able to increase fonts. I also might move this to lower in the figure since you present actual data on the fetal components in 1C, and 1B could be a nice summary of the overlaps between the fetal and tHIOs timepoints if you moved it to the bottom.

We increased the text size in this figure and moved it to the end of Figure 1 as a summary.

6. Fig 1C and 1D. Can you indicate which muscle layer is being newly formed in each image since there are several highlighted in the figure?

We added the description of muscle layer formation over time to the figure caption.

7. Fig. 2C is it difficult to appreciate the olfm4 localization at this magnification, might be useful to use a higher magnification to highlight the crypts.
We have added insets to the figure to highlight some of the crypts.

© 2023. Published by The Company of Biologists under the terms of the Creative Commons Attribution License (https://creativecommons.org/licenses/by/4.0/).

8. Fig 2D quantification of the Ki67 expression per region of the epithelium would be a useful adjunct.

We have added quantification of percent of epithelial MKi67+ cells that are in the crypt to the Supplement.

9. figs 3C and 3C - difficult to read the labels at this font size. please increase size or add a legend.

We have increased the size of the font in these images.

10. Fig 4. I would like to see D and E enlarged to make it easier to appreciate the data. F and G could be move to a supplementary figure if needed. I would also add a header for F & G saying reference vs tHIO because you can't tell what you are looking at without referring to legend.

We have enlarged D and E and moved F and G to Supplemental Figure 3.

11. Supp Fig 1C - the Alpi staining appears to just be picking up the mucin layer, I am not seeing clear data that show it is staining enterocytes.

We recognize the concern, as we do not wish to show background staining from the mucin layer. For this reason, we used the Vector Red Substrate Kit-Alkaline Phosphatase to stain for ALPI activity rather than an antibody for the protein. Thus, we believe that the images in S1C represent functional protein rather than background staining. Additionally, ALPI does not appear to be staining the insides of the goblet cells, which we normally see in background stains. Notably, our ALPI expression appears to be similar to what is seen in the Human Protein Atlas, which also shows staining at the tips of the epithelial cells (https://www.proteinatlas.org/ENSG00000163295-ALPI/tissue/small+intestine#img). We have added further clarification in the "Materials and Method Section".

12. Supp Fig 2 - please increase label sizes or add a legend.

We have increased the size of the font in these images.

13. Supp Fig 3 - this is one of the most interesting components of the paper given the inherent difficulties in studying mesenchymal development and an advantage of this model system. I would consider moving it out of the supplement if space allows. Would also make space changes as suggested for the similar figure 4 as remarked in item 10 above.

Thank you for this suggestion; we definitely agree that the mesenchymal analysis is incredibly important and a major advantage of tHIOs. Unfortunately, due to space constraints, we were unable to move this analysis out of the supplement. We were able to make the space changes as suggested, however.

Minor points:
page 9 line 1 - HIOs were transplanted

Thank you for finding this error; it has been corrected.

page 23 bottom "Our findings suggest that tHIOs are a fantastic proxy for studying the development of the human fetal intestine." I would consider removing the word fantastic.

We have removed 'fantastic' from the sentence.

Reviewer 2 Advance Summary and Potential Significance to Field...

For ethical and technical reasons, the spatiotemporal regulation of human intestinal development has been elusive. Singh et al. have carried out temporal single-cell transcriptomic analysis for transplanted human intestinal organoids and provided a valuable resource to understand human intestinal development. The authors employed transplanted human intestinal organoids, which recapitulate human intestinal development and thoroughly analyzed their transcriptomes at a

© 2023. Published by The Company of Biologists under the terms of the Creative Commons Attribution License (https://creativecommons.org/licenses/by/4.0/).

single-cell level over time. Because this paper was submitted as a technical resource section, there are few novelties in the article.

However, the dataset is instructive for the community and will be suitable for Development if the authors address the following points.

Reviewer 2 Comments for the Author...

1. Although I understood this paper is in the resource paper format, the Results and Discussion sections are mainly descriptive. The authors should try to provide some analyses that could deepen the regulation of human intestinal development. For instance, receptor-ligand analysis using scRNA/snRNAseq data would be helpful. Which mesenchymal subpopulation expresses important niche factors, such as Wnt2b?

Thank you for this suggestion. We have completed a receptor/ligand analysis for *WNT2B*, *WNT5A*, and *NRG1*, and added these findings to the Results & Discussion section.

2. Related to the above point, telocytes, one of the mesenchymal subpopulations, was reported to express Wnt niche factors for stem cell self-renewal. Yet, later reports have challenged this concept (McCarthy N et al. Cell Stem Cell 2020). Because these data are mainly derived from adult intestines, it would be interesting to delineate the Wnt and other niche factor expression profiles in the developmental mesenchymal subpopulations.

Thank you for this suggestion. We completed this analysis concurrently with the response to Point 1.

3. Page 17. The authors indicated “the emergence of intestinal function.” However, I am not sure this description is appropriate because they only provided functional marker expression data. “Function” implies intestinal digestion and absorption function, but simple gene/protein expression might be insufficient to conclude the biological function.

Thank you for identifying this concern. We have changed “intestinal function” to “cellular maturation” to reflect this.

4. The authors showed poor efficient recovery of mesenchymal cells in their scRNA-seq analysis compared with snRNA-seq. However, the subpar recovery was simply due to the choice of digestive enzyme. According to their method sections, they used TrypLE Select to dissociate tHIOs for scRNAseq analysis. Because mesenchymal cells are embedded in ECM, including trypsin-resistant collagen, their method favors isolating epithelium but not mesenchyme. I would use trypsin for epithelial isolation and collagenase or other ECM degrader to isolate mesenchymal cells. It is probably unrealistic to redo the experiments, and the authors should include this possibility in their paper.

Thank you for this insight. We have added this to the “limitations” paragraph in the results section. We did attempt to dissociate tHIOs using an enzyme cocktail that included multiple collagenases, but cell death rate was high and the yield of non-epithelial cells never exceeded 20% of the live cells.

5. Yap signal is activated in the mouse embryonic intestines. The authors focused on LGR5 stem cells, but it would be better to include YAP staining/YAP gene signature analysis during human intestinal development and tHIO development.

Thank you for this suggestion. We have analyzed YAP gene signature and YAP-TAZ protein expression in both fetal human intestine as well as in tHIOs, and have included the results in the manuscript.

© 2023. Published by The Company of Biologists under the terms of the Creative Commons Attribution License (https://creativecommons.org/licenses/by/4.0/).

Second decision letter

MS ID#: DEVELOP/2022/201416

MS TITLE: Transplanted Human Intestinal Organoids: A Resource for Modeling Human Intestinal Development

AUTHORS: Akaljot Singh, Holly M Poling, Praneet Chaturvedi, Konrad Thorner, Nambirajan Sundaram, Daniel O. Kechele, Charlie J. Childs, Heather A. McCauley, Garrett W. Fisher, Nicole E Brown, Jason R. Spence, James M Wells, and Michael Helmrath
ARTICLE TYPE: Techniques and Resources Report

I am happy to tell you that your manuscript has been accepted for publication in Development, pending our standard ethics checks.

Reviewer 1

Advance summary and potential significance to field

The authors submit a, now revised, manuscript detailing the longitudinal developmental sequence of tHIOs and their relevance to human fetal development.

Comments for the author

The authors have satisfactorily addressed all of my previous concerns/comments.

Reviewer 2

Advance summary and potential significance to field

The authors appropriately responded to my comments and significantly improved the quality of the manuscript. I think the paper is now ready for publication.

Comments for the author

None

© 2023. Published by The Company of Biologists under the terms of the Creative Commons Attribution License (https://creativecommons.org/licenses/by/4.0/).
